# Predictive Approach Identifies Molecular Targets and Interventions to Restore Angiogenesis in Wounds With Delayed Healing

**DOI:** 10.3389/fphys.2019.00636

**Published:** 2019-05-28

**Authors:** Sridevi Nagaraja, Lin Chen, Luisa A. DiPietro, Jaques Reifman, Alexander Y. Mitrophanov

**Affiliations:** ^1^Department of Defense, Biotechnology High Performance Computing Software Applications Institute, Telemedicine and Advanced Technology Research Center, United States Army Medical Research and Materiel Command, Fort Detrick, MD, United States; ^2^The Henry M. Jackson Foundation for the Advancement of Military Medicine, Inc., Bethesda, MD, United States; ^3^Center for Wound Healing and Tissue Regeneration, College of Dentistry, University of Illinois at Chicago, Chicago, IL, United States

**Keywords:** wound healing, angiogenesis, endothelial cells, computational analysis, vascular endothelial growth factor

## Abstract

Impaired angiogenesis is a hallmark of wounds with delayed healing, and currently used therapies to restore angiogenesis have limited efficacy. Here, we employ a computational simulation-based approach to identify influential molecular and cellular processes, as well as protein targets, whose modulation may stimulate angiogenesis in wounds. We developed a mathematical model that captures the time courses for platelets, 9 cell types, 29 proteins, and oxygen, which are involved in inflammation, proliferation, and angiogenesis during wound healing. We validated our model using previously published experimental data. By performing global sensitivity analysis on thousands of simulated wound-healing scenarios, we identified six processes (among the 133 modeled in total) whose modulation may improve angiogenesis in wounds. By simulating knockouts of 25 modeled proteins and by simulating different wound-oxygenation levels, we identified four proteins [namely, transforming growth factor (TGF)-β, vascular endothelial growth factor (VEGF), fibroblast growth factor-2 (FGF-2), and angiopoietin-2 (ANG-2)], as well as oxygen, as therapeutic targets for stimulating angiogenesis in wounds. Our modeling results indicated that simultaneous inhibition of TGF-β and supplementation of either FGF-2 or ANG-2 could be more effective in stimulating wound angiogenesis than the modulation of either protein alone. Our findings suggest experimentally testable intervention strategies to restore angiogenesis in wounds with delayed healing.

## Introduction

**FIGURE 1:**
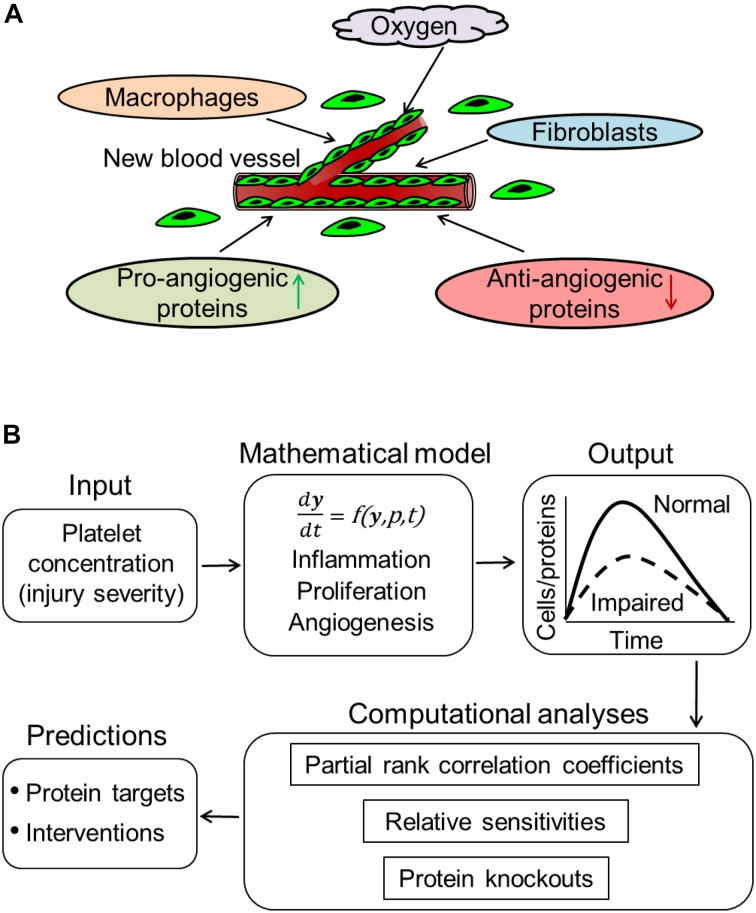
Computational modeling of angiogenesis in wound healing. **(A)** Low oxygen levels and wound proteins released at the wound site by inflammatory (e.g., macrophages) and proliferative (e.g., fibroblasts) cells induce the migration of ECs from surrounding blood vessels into the wound. These ECs proliferate at the wound site, release angiogenic proteins, and organize into capillary sprouts. Signaling by both pro- and anti-angiogenic proteins prompts the capillary sprouts to undergo elongation, branching, regression, and anastomosis with existing or newly formed blood vessels. **(B)** Our computational model describes an injury-initiated wound-healing response. The model captures inflammation, proliferation, and angiogenesis during wound healing. The model describes the kinetics of platelets, 9 cell types, 29 proteins, oxygen, and 133 biological processes. In our model, the rates of the various biological processes are represented by 159 model parameters. The model simulates the time courses of these 40 variables across a 42-day period post-wounding during normal and impaired angiogenesis in wounds. We performed various computational analyses, including calculation of partial rank correlations coefficients, calculation of sensitivities, and simulating protein knockouts, to predict protein targets and intervention strategies to restore angiogenesis in wounds. These analyses are described in the “Materials and Methods” Section.

Impaired angiogenesis, a typical phenotype of non-healing wounds, is predictive of delayed wound healing (Demidova-Rice et al., 2012; Wietecha and DiPietro, 2013; Bodnar, 2015; Okonkwo and DiPietro, 2017). Normal angiogenesis is essential for the delivery of immune cells, nutrients, and oxygen to promote the regeneration of granulation tissue in a wound (Demidova-Rice et al., 2012; Yoo and Kwon, 2013; Okonkwo and DiPietro, 2017). If left untreated, impaired angiogenesis can lead to serious pathologies, such as wound ischemia and chronicity, as seen in traumatic skin injuries (e.g., burn wounds) and in the wounds of diabetics (e.g., diabetic ulcers) (Sun et al., 2011; Demidova-Rice et al., 2012; Sorg et al., 2017). Given the increasing rate of diabetes in the general population, there is a pressing need for effective treatments to restore wound angiogenesis (Tang et al., 2013). However, the complex and dynamic nature of angiogenesis poses a significant challenge to the development of efficacious therapeutic interventions. Angiogenesis involves numerous biological processes influenced by the cellular signaling that occurs during different phases of wound healing (e.g., the inflammatory and proliferative phases) (Figure 1A) (Yoo and Kwon, 2013; Sorg et al., 2017). A mechanistic understanding of the molecular and cellular processes involved in angiogenesis, particularly of angiogenic protein signaling, may accelerate ongoing efforts to identify new and efficacious interventions to restore angiogenesis in wounds with delayed healing, such as diabetic and burn wounds.

Current therapies used to restore angiogenesis in wounds, such as tissue-engineered dressings, hyperbaric oxygen, and negative pressure, are often inadequate (Wietecha and DiPietro, 2013; Okonkwo and DiPietro, 2017). The modulation of wound proteins (chemokines, cytokines, and growth factors) is regarded as a promising approach to stimulate angiogenesis and is the focus of many ongoing efforts (Yoo and Kwon, 2013; Bodnar, 2015; Treps et al., 2016). For instance, vascular endothelial growth factor (VEGF)—a known pro-angiogenic protein—is produced by the majority of cell types involved in innate immune signaling (including macrophages, fibroblasts, and keratinocytes) and is significantly lowered in the wounds of diabetic mice compared to those of healthy mice (Altavilla et al., 2001; Kampfer et al., 2001; Hoffman et al., 2006; Okizaki et al., 2016). Therefore, from a preclinical perspective, VEGF is an ideal factor for angiogenesis stimulation in wounds, and its supplementation has been the focus of many investigations (Barrientos et al., 2014; Johnson and Wilgus, 2014). However, in a phase II clinical trial, topical VEGF failed to improve diabetic foot ulcer healing (Barrientos et al., 2014; Okonkwo and DiPietro, 2017). Furthermore, interventions involving the topical application of platelet-derived growth factor, supplementation of fibroblast growth factor-2 (FGF-2), or inhibition of transforming growth factor (TGF)-β to stimulate angiogenesis have previously been tested in pathological wounds with limited success (Ramsauer and D’Amore, 2007; Liu et al., 2009; Barrientos et al., 2014). Recently, interventions with newer protein targets have emerged; for example, modulation of the chemokines CXCL8 and CXCL12 or their receptors—either individually or in combination—has been investigated as a novel therapeutic option for treating impaired angiogenesis (Bodnar, 2015). However, given the large number of proteins that participate in wound healing and their multifunctional roles in its different phases, it is extremely challenging to predict the effects of their modulation *in vivo* using qualitative intuition alone.

Computational modeling approaches can complement traditional experimental approaches in the search for promising therapeutic targets and optimal intervention strategies to restore angiogenesis by systematically analyzing thousands of wound-healing scenarios in a non-reductionist, system-focused framework. Computational models representing angiogenesis *per se* in wounds have been developed (Logsdon et al., 2014; Flegg et al., 2015). However, existing models are limited in their ability to capture the interactions between the molecular and cellular processes involved in the different phases of wound healing (i.e., inflammation, proliferation, and angiogenesis), as well as in their ability to capture the effects of inflammatory and proliferative proteins on angiogenesis. The goals of this study are threefold: (1) to develop a quantitative kinetic model of wound healing that captures inflammation, proliferation, and angiogenesis in wounds, (2) to use this model to predict influential cellular and molecular processes, as well as protein targets, for angiogenesis regulation, and (3) to further use the model to predict optimal intervention strategies to restore angiogenesis in wounds with delayed healing. To achieve these goals, we extended our computational model of wound inflammation and proliferation (Nagaraja et al., 2017) to represent angiogenesis during wound healing (Figure 1). Using this extended model, we simulated wound-healing scenarios with normal or impaired (particularly, decreased) angiogenesis. Specifically, we focused on wounds with decreased levels of endothelial cells (ECs) and VEGF because they are typically observed in wounds with delayed healing (e.g., diabetic wounds) (Altavilla et al., 2001; Kampfer et al., 2001; Hoffman et al., 2006; Okizaki et al., 2016).

Our analysis of 60,000 model-simulated wound-healing scenarios identified six (among the 133 modeled) influential molecular and cellular processes for angiogenesis regulation in wounds, as follows: VEGF degradation, TGF-β degradation, fibroblast apoptosis, fibroblast migration, EC migration, and EC apoptosis. Next, we identified oxygen, as well as four of the 29 modeled proteins [namely, TGF-β, VEGF, FGF-2, and angiopoietin-2 (ANG-2)], as potential targets whose modulation may increase angiogenesis in wounds with delayed healing. Third, our results suggested that angiogenesis and collagen deposition during wound healing can be improved by (1) the lowering of either TGF-β or oxygen levels in wounds and (2) the supplementation of wounds with either FGF-2 or ANG-2 individually. Interestingly, ANG-2 is a known regulator of angiogenesis (Yoo and Kwon, 2013) while VEGF, FGF-2, and TGF-β have been tested individually as therapeutic agents to improve wound-healing outcomes in past clinical trials with limited success (Hanft et al., 2008; Ferguson et al., 2009; Logsdon et al., 2014). A plausible reason for this lack of success is that the effects of supplementing these proteins had been anticipated without considering the relevant mechanistic context (i.e., interactions among different wound-healing phases). The level of mechanistic detail in our model enabled the investigation of single-protein modulation while accounting for redundancies in protein functions and for the multifaceted roles of single proteins. For example, our intervention simulations demonstrated that modulation of single proteins (e.g., TGF-β) improved angiogenesis to certain extent, but did not resolve delayed wound closure. Our model thereby facilitates a complementary approach to study the effects of new therapies on multiple wound-healing endpoints, expanding the pool of proteins that could serve as potential therapeutic targets. Finally, our results support the growing consensus that modulation (i.e., inhibition or supplementation) of two or more protein targets (or a protein and oxygen) is more efficacious in restoring angiogenesis than modulation of either target alone (Gerber et al., 1998; Arsic et al., 2003; Liu et al., 2009; Allen et al., 2014; Gao et al., 2015). Our results are corroborated by existing experimental data and suggest new intervention strategies that can be experimentally tested.

## Materials and Methods

### Computational Model of Wound Healing

The computational model presented in this study is an extension of our recently developed model of injury-induced wound healing (Nagaraja et al., 2017). That wound-healing model describes the kinetics of platelets, four inflammatory cell types (namely, pro- and anti-inflammatory macrophages, active neutrophils, and apoptotic neutrophils), two proliferative cell types (namely, fibroblasts and myofibroblasts), 18 wound proteins (cytokines and growth factors), and three forms of collagen. It also reflects essential interactions of these proteins and cell types during normal healing and pathological scarring in traumatic injuries. To that model, we added mathematical descriptions of the kinetics of ECs, blood vessels, keratinocytes, eight angiogenic proteins not included in our previous model [namely, VEGF, angiopoietin (ANG)-1 & 2, thrombospondin (TSP)-1, endostatin, chemokine CXCL1, pigment epithelium-derived factor (PEDF), and keratinocyte growth factor (KGF)], and oxygen. We modeled these components because they are commonly regarded as cell types and molecules essentially involved in angiogenesis (Figure 1). The current version of the model describes the kinetics of 40 variables. The model is a coupled system of 39 ordinary differential equations and one delay differential equation, where each equation describes the kinetics of one model variable (Supplementary Table S2). Each model variable represents the volumetric concentration of a given molecular or cellular species at a given time. We modeled 133 different molecular and cellular processes, such as chemotaxis of different modeled cell types, cellular proliferation, and the production and degradation of various wound proteins. These processes, listed in Supplementary Table S1, are characterized by 159 model parameters; some processes required more than one parameter to describe their kinetics. The default values, units, and descriptions of these 159 parameters, as well as the specific processes each represents, are given in Supplementary Table S2.

Whenever published data from cell-culture experiments were available, we used them to derive the values of the production and degradation rates for different wound proteins, as previously described (Nagaraja et al., 2014). As an illustration, Supplementary Figure S1A shows the endostatin production rate estimation from experimental data in cultured ECs using linear regression. To approximate the kinetics of EC chemotaxis induced by VEGF, we fit available data using a quadratic equation with two parameters (Supplementary Figure S1B). In addition to inducing chemotaxis, wound proteins provide regulatory feedback during angiogenesis by upregulating (positive feedback) or downregulating (negative feedback) the production of other proteins, cell proliferation, or cellular apoptosis. In our model, we included both pro-angiogenic proteins, such as VEGF and ANG-1&2, and anti-angiogenic proteins, such as TSP-1, PEDF, and endostatin. To represent the pro- and anti-angiogenic effects of these proteins, we introduced 18 dimensionless feedback functions (see Supplementary Table S2) that represented fractional increases or decreases (induced by a particular protein) in the biological activities of other cells or proteins. The parameters in these functions supplement the 159 default parameters described above. We estimated the parameters of these functions by fitting linear, quadratic, or polynomial functions to experimental data using MS EXCEL (Supplementary Figures S1C,D).

We simulated the time courses for each of the 40 model variables over a 42-day period after wounding. We chose day 42 as the final time point for our simulations because we assumed it to be representative of the time required for the completion of the proliferative phase of wound healing, which results in scar formation (Gauglitz et al., 2011). The simulation performed using the default parameter set represented the cellular and molecular time courses during a normal-healing response to injury. We subsequently modified the default parameter set to simulate 60,000 unique wound-healing scenarios. In addition, we simulated one specific impaired-angiogenesis scenario by modifying the default values of only two of the model parameters (see the next subsection).

During model development, we made simplifying assumptions regarding the complex, multi-step angiogenesis process, comprising EC migration, vessel sprouting and branching, anastomosis, vessel regression, and vessel stabilization. For example, given a lack of sufficient mechanistic data, we assumed that all ECs migrating to the wound site form capillary tips, and that these tips undergo anastomosis to form blood vessels. Moreover, we assumed that newly formed blood vessels are fully functional. In reality, early vessels are leaky and tortuous, and they undergo significant refinement during wound remodeling. Eventually, the remaining vessels are stabilized by pericytes that migrate to the wound site during vessel regression. While we did not explicitly model the migration of pericytes and their role in vessel regression, we modeled the effect of anti-angiogenic proteins, such as PEDF and TSP-1, on EC migration and EC apoptosis (which contribute to vessel regression) (Short et al., 2005; Longeras et al., 2012).

We performed all simulations in the software suite MATLAB R2017b (MathWorks, Natick, MA, United States) and solved the model equations using the MATLAB solver DDE23 with default tolerance levels. We used this model with different computational strategies to identify (1) the molecular and cellular processes (among the 133 represented) whose modulation strongly influences angiogenesis, (2) protein targets to regulate angiogenesis in wounds, and (3) therapeutic interventions to restore angiogenesis in wounds with delayed healing. These computational strategies are described below.

### Identification of Influential Molecular and Cellular Processes for Angiogenesis

To identify the molecular and cellular processes that are most influential for angiogenesis regulation, we used two computational methods—global sensitivity analysis (GSA) and extended local sensitivity analysis (LSA)—to quantify the influence of each of the 159 model parameters on two specific model variables, namely, the concentrations of ECs and VEGF. We assumed that the strength of influence of a model parameter reflected the influence of the corresponding biological process whose rates are governed by that parameter. We chose to focus on the EC and VEGF model variables as end-point readouts because they are regarded as reliable indicators of the angiogenesis level in a wound (Wietecha and DiPietro, 2013; Yoo and Kwon, 2013). First, we used Latin hypercube sampling (MATLAB function LHSDESIGN) to create 60,000 unique parameter sets (Nagaraja et al., 2014, 2017). The parameter values in these sets were randomly selected from a 5-fold interval (up to 2.5-fold variations above or below the default value). This random sampling was intended to represent possible natural variations in the wound-healing process. Our choice of interval reflected the experimentally observed variability of these processes in mice (Nagaraja et al., 2017). Next, using each parameter set, we simulated 60,000 distinct wound-healing scenarios. In each of these scenarios, we simulated the time course for each of the 40 model variables over a 42-day period post-wounding (Figure 1B). However, among the 60,000 simulations, 8,825 did not converge in a pre-defined time period (1 min) even after implementing the smallest time step (1 × 10^−12^) allowed by the MATLAB solver used in our simulations (i.e., DDE23s). This lack of convergence within the specified time interval indicated that these simulations had significant irregularities, suggesting that their kinetic behavior was biologically unrealistic. Therefore, we excluded these 8,825 simulations from further analysis. Thus, we used 51,175 simulations and their corresponding parameter sets for the GSA and the extended LSA.

For the GSA, we calculated the partial rank correlation coefficients (PRCCs) between the model parameter values and the VEGF and EC concentrations. Using the values of the model variables from the 51,175 simulations and the model parameter values from the corresponding parameter sets, we separately calculated the Spearman PRCCs (with their associated *P*-values; MATLAB function PARTIALCORR) between each of the two model variables (i.e., VEGF or EC) and each of the 159 model parameters at 42 time points, where each point represented a post-wounding day. We specifically chose to calculate the Spearman PRCCs because they provide a measure of the strength of monotonic dependence between a model parameter and a model variable, while eliminating the effects of the dependence of any given variable on other variables in the system (e.g., the dependence of VEGF on the variables representing macrophages and fibroblasts) (de la Fuente et al., 2004). The Spearman PRCCs vary between −1 and +1 and the sign of the PRCC values indicated the positive or negative directionality of the correlation between a model parameter and a model variable. A PRCC with *P* ≤ 0.05 indicated that it was statistically significantly different from zero. As a result of our PRCC analysis, we obtained 159 PRCCs for EC and 159 PRCCs for VEGF. The model parameters whose absolute values of the PRCCs with the EC or the VEGF variables were greater than 0.5 with *P* ≤ 0.05 were regarded as influential for that variable. The biological processes represented by those parameters were regarded as the strongest influencers for angiogenesis regulation.

For the extended LSA, we calculated the logarithmic (i.e., the relative) sensitivity of the EC and VEGF model variables with respect to each of the 159 model parameters in each of the 51,175 simulations (Mitrophanov et al., 2007). We have previously used this method to determine the mechanistic drivers of chronic inflammation and pathological scarring in wounds (Nagaraja et al., 2014, 2017). Briefly, for each of the 51,175 parameter sets, we individually perturbed each parameter by 10% of its default value in that particular set, and calculated the corresponding relative change in the EC and VEGF model variables using the second-order central finite difference formula. The absolute values of the sensitivities indicated the strength of influence of a given model parameter on a given model variable. As a result of our LSA, we obtained 51,175 local sensitivity values for each parameter-variable combination. For each simulation, we sorted the absolute values of the sensitivities of EC and VEGF to the 159 model parameters in descending order. Next, for both EC and VEGF, we calculated the percentage of the 51,175 simulations (or wound-healing scenarios) for which their sensitivity to each model parameter ranked at the top. The model parameters that ranked at the top in the majority of the 51,175 simulations (for either the EC or the VEGF variable) were regarded as the parameters exerting the strongest influence on a given model variable. We combined the results from the PRCC and extended local sensitivity analyses to arrive at the final list of the most influential model parameters for ECs and VEGF. The biological processes represented by these model parameters were considered the most influential processes for angiogenesis regulation.

From this list of the model-identified parameters that were influential for angiogenesis regulation, we modified the default values of two model parameters (while keeping the remaining ones at their default values) to simulate one specific impaired-angiogenesis scenario characterized by decreased levels of VEGF and ECs, as seen in wounds with delayed healing (Streit et al., 2000; Hoffman et al., 2006; Galeano et al., 2008; Mirza et al., 2009). The modified values of the two parameters (reflecting angiogenesis impairment) were chosen by simultaneously fitting our model simulations for VEGF and EC to the corresponding data from the wounds of diabetic mice (known to exhibit impaired angiogenesis) (Supplementary Figures S1E,F) (Altavilla et al., 2001; Kampfer et al., 2001; Hoffman et al., 2006; Okizaki et al., 2016). This resulted in a 3-fold decrease in the default value of the rate of VEGF production by anti-inflammatory macrophages, and a 1.2-fold increase in the default value of the EC apoptosis rate. We subsequently used this impaired-angiogenesis simulation obtained with these changed parameter values to investigate the model-predicted interventions to restore angiogenesis.

### Protein-Knockout (KO) Analysis

To identify proteins (among the 29 modeled) that could be promising therapeutic targets to stimulate angiogenesis in wounds with delayed healing, we performed (simulated) protein KO analysis. For this analysis, we used the data from the 51,175 wound-healing simulations performed for the GSA. For each simulation, we calculated the fold changes in the peak concentrations of ECs and VEGF relative to their respective peak concentrations in the simulation with the default parameter set. Based on these fold changes, we classified the 51,175 simulations into two groups. Simulations for which both EC and VEGF fold changes were <0.2 were classified as “impaired angiogenesis.” Those for which the fold changes were simultaneously ≥0.8 and ≤1.5 were classified as “normal angiogenesis.” If the fold changes did not satisfy the cutoff values for either classification, then the results from such simulations were not included in further analyses. The fold-change cutoff values for determining angiogenesis impairment were chosen based on the observed decrease in VEGF and EC levels in diabetic wounds (Altavilla et al., 2001; Kampfer et al., 2001; Hoffman et al., 2006; Okizaki et al., 2016). This step in the classification process was performed to ensure that the simulated wound-healing scenarios classified as “impaired angiogenesis” demonstrated the experimentally observed symptoms (i.e., VEGF and EC deficiencies) of impaired angiogenesis in wounds. Next, we filtered out some simulations from within the “normal” and “impaired” angiogenesis groups based on the values of two specific model parameters—namely, the rate of VEGF production by anti-inflammatory macrophages and the EC apoptosis rate. Among the simulations classified as “normal angiogenesis,” only simulations for which the VEGF production rate was >2 × 10^−7^ and the EC apoptosis rate was <0.099 were retained in the “normal angiogenesis group.” Similarly, within the “impaired angiogenesis” group, we retained only the simulations for which the parameter value for the VEGF production rate was ≤2 × 10^−7^ and the EC apoptosis rate was ≥0.099. We performed this second classification step to ensure that the simulations in the “impaired angiogenesis” group not only captured the symptoms of angiogenesis, but also demonstrated the mechanistic disruption underpinning the angiogenesis impairment, i.e., dysregulation in the VEGF production and EC apoptosis (because these processes were identified as influential for angiogenesis regulation in the GSA and the extended LSA). Similarly, we sought to ensure that the rates of these processes were in the vicinity of their default values for the simulations classified as “normal angiogenesis.”

Next, we simulated the KO of 25 of the 29 modeled proteins (one at a time), as well as modification of oxygen levels, in each of the simulations present in the “impaired angiogenesis” group (after the second classification step). We did not simulate the KO of KGF and the three forms of collagen included in the model because they are structural proteins comprising the extracellular matrix and granulation tissue and are required for wound closure. We simulated the protein KO of each of the 25 modeled proteins by modifying the parameter sets of the “impaired angiogenesis” group simulations. Specifically, in each of those sets, we changed the values of the parameters representing the production rates of a given protein or the rate of oxygen release from blood vessels to zero. Finally, we compared the means and standard deviations of the EC and VEGF peak concentrations between the simulations from the 28 groups, i.e., “normal angiogenesis,” “impaired angiogenesis,” 25 “impaired angiogenesis with protein KO,” and the “impaired angiogenesis with oxygen level modification” groups. The proteins for which the KO increased or decreased (*P* ≤ 0.05) the mean values of EC or VEGF concentrations (compared to the simulations from the impaired angiogenesis group) were identified as potential targets for therapeutic interventions.

### Protein Inhibitor Modeling

Based on the results of the PRCC, extended LSA, and protein KO analyses, we simulated different interventions aimed at increasing angiogenesis levels in wounds with delayed healing. For the proteins whose KO increased the peak VEGF or EC concentration in impaired-angiogenesis simulations (see previous paragraph) with *P* ≤ 0.05, we explicitly modeled their inhibitors. We accomplished this by adding two equations that represented two new variables (in addition to the 40 variables in the default model)—a protein inhibitor and the inhibitor-protein complex (see Supplementary Table S2). We also added two new model parameters that represented the association (k_on_) and dissociation (k_off_) rate constants for the protein and its inhibitor (P#160 and P#161, see Supplementary Table S2). We derived the values of these parameters from available experimental data (De Crescenzo et al., 2003; Nagaraja et al., 2015). We simulated the addition of the inhibitors at different concentrations and at two separate time points, i.e., 1 h and 24 h post-wounding. For the proteins whose KO decreased the peak VEGF or EC concentrations in the “impaired angiogenesis” group with *P* ≤ 0.05, we simulated interventions which involved supplementation of those proteins at different concentrations and at different times post-wounding. To this end, we introduced two new model parameters that represented the concentration doses of the proteins being supplemented (Supplementary Table S2). We simulated the supplementation of the proteins at two different concentrations at two separate time points, i.e., 1 h and 24 h post-wounding. Finally, we simulated interventions that involved simultaneous inhibition or supplementation of single or multiple proteins to identify the most efficacious interventions to restore angiogenesis in wounds with delayed healing.

## Results

### Computational Model Captures Normal and Impaired Angiogenesis Dynamics in Wounds

**FIGURE 2:**
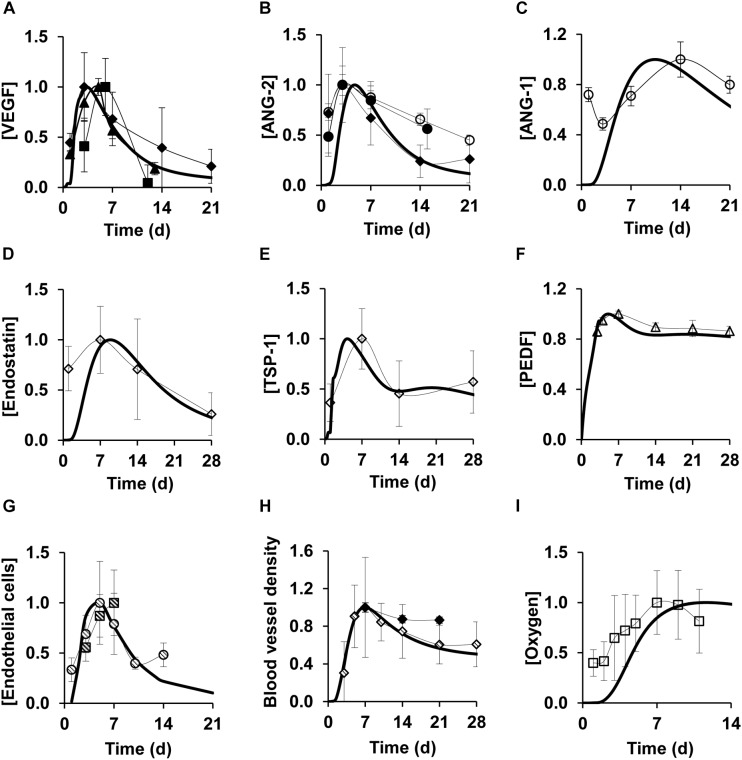
Model simulations capture experimentally detected time courses of normal angiogenesis in skin wounds. Solid lines show model simulations; symbols show experimental data. Brackets designate normalized concentrations of **(A)** VEGF, **(B)** ANG-2, **(C)** ANG-1, **(D)** endostatin, **(E)** TSP-1, **(F)** PEDF, **(G)** endothelial cells, **(H)** blood vessel density, and **(I)** oxygen. Day 0 indicates the day of injury initiation. Experimental data were obtained from previously published experimental studies in mouse wounds and dog wounds, eye, and cardiac tissue in mice: filled circles (*N* = 22) (Ma et al., 2016), filled diamonds (*N* = 6) (Matsunaga et al., 2003), filled triangles (*N* = 12) (Schiefelbein et al., 2008), filled squares (*N* = 7) (Altavilla et al., 2001), open circles (*N* = 8) (Zhao et al., 2014), open diamonds (*N* = 6) (Olenich et al., 2014), open triangles (*N* = 4) (Renno et al., 2002), open squares (*N* = 12) (Desmet et al., 2015), dashed circles (*N* = 5) (Zhao et al., 2016), dashed diamonds (*N* = 8) (Wietecha et al., 2015), and dashed squares (*N* = 6) (Okizaki et al., 2016). For proper comparisons between model predictions and experimental data, normalization was necessary because of the differences in reporting units between experimental data and model simulations. For each model-predicted time course, normalization was performed by dividing that time course by its maximal value. For each time course obtained from available experimental data, we first extracted the means and standard deviations (based on the sample size information provided in each study) at each time point. Then, we divided the mean and standard deviation values by the maximal mean value in the time course.

We tested the ability of our model to capture the typical features of the angiogenesis response by comparing our model simulations of the time courses for different cell types, proteins, and oxygen with published experimental data (these validation data sets were not used in developing our model). In our simulations, we detected a peak in the levels of pro-angiogenic proteins—such as VEGF and ANG-2—between days 4 and 5 post-wounding (Figure 2A,B) while ANG-1 levels peaked around day 10 (Figure 2C). The EC concentration peaked near day 6 (Figure 2G). These predictions matched the corresponding measurements in mouse wounds (Altavilla et al., 2001; Schiefelbein et al., 2008; Zhao et al., 2016), rat wounds (Zhao et al., 2014), human wounds (Ma et al., 2016), as well as those from an experimental study of myocardial ischemia in dogs (Matsunaga et al., 2003). In the prior experiments, EC expression levels were quantified by staining the wounds with an antibody against the EC marker CD31 and measuring the percent area of wound bed occupied by CD31 staining (Matsunaga et al., 2003; Mirza et al., 2009). Anti-angiogenic proteins endostatin, TSP-1, and PEDF peaked between days 5 and 12 and did not completely return to their baseline levels by day 28 post-wounding (Figure 2D,F). These predictions matched the corresponding measurements in the wounds of mice and retinal wounds in rats (Renno et al., 2002; Olenich et al., 2014). The EC concentration, however, returned to its baseline level near day 21 (Figure 2G). In the model, ECs reflect the concentrations of newly formed capillary tips that eventually undergo anastomosis with other capillary tips and with existing or newly formed blood vessels. Our simulations showed that the blood vessel density peaked shortly after the EC peak (around day 7) and began to stabilize around day 28 post-wounding (Figure 2H). The predictions for blood vessel density matched the respective experimental measurements in mice (Matsunaga et al., 2003; Wietecha et al., 2015). In the prior experiments, the blood vessel densities were determined by counting the vessel profiles (rounded or elongated spaces bounded by CD31-staining ECs) per unit area (Hoffman et al., 2006; Okizaki et al., 2016).

The majority of cutaneous wounds are initially hypoxic due to loss of existing blood vessels at the time of wounding (Darby and Hewitson, 2016). Oxygen levels in a wound are restored as new blood vessels emerge. Our model simulations of wound oxygen concentration showed that the oxygen level peaked near day 10 (Figure 2I), shortly after the blood vessel density peaked at approximately day 7. The oxygen levels remained steady after reaching the peak, as the newly formed blood vessels re-established perfusion in the wound. In all our comparisons (Figure 2), the model-predicted time courses for the angiogenic cell types and proteins showed reasonably good agreement with experimental data. Indeed, for the majority of the comparisons, our model simulations lay within ±1 standard deviation of the normalized experimental data. That pattern was present despite the fact that quantitative comparisons were hampered by differences between the units used to report the experimental measurements and the (absolute) units in our model simulations before data normalization.

**FIGURE 3:**
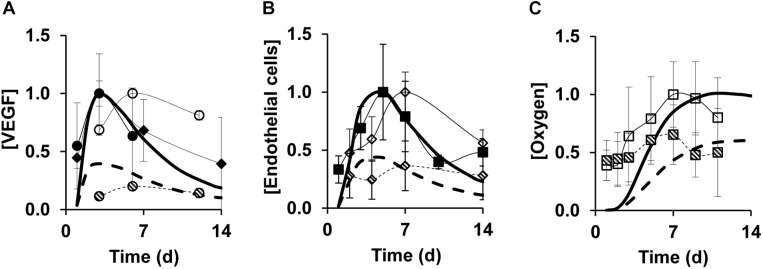
Model predictions capture experimentally detected time courses of normal and impaired angiogenesis in skin wounds. Solid lines show model simulations for normal angiogenesis and dashed lines show model simulations for impaired angiogenesis. Solid and open symbols show experimental data from the wounds of wild-type mice and dashed symbols show experimental data from the wounds of diabetic mice. Brackets designate normalized concentrations of **(A)** VEGF, **(B)** endothelial cells, and **(C)** oxygen. Day 0 indicates the day of injury initiation. Experimental data were obtained from previously published experimental studies in mouse wounds: open and dashed circles (*N* = 7) (Galeano et al., 2008), open and dashed squares (*N* = 14 and 12, respectively) (Desmet et al., 2015), open and dashed diamonds (*N* = 5) (Streit et al., 2000), solid circles (*N* = 6) (Ishida et al., 2004), solid squares (*N* = 8) (Wietecha et al., 2015), and solid triangles (*N* = 6) (Matsunaga et al., 2003). Impaired angiogenesis in the computational model was induced by decreasing the rate of VEGF production by anti-inflammatory macrophages by 3-fold and increasing the EC apoptosis by 1.2-fold from their respective default values. For proper comparisons between model predictions and experimental data, normalization was necessary because of the differences in reporting units between experimental data and model predictions. For each model-predicted time course, normalization was performed by dividing that time course by the maximal value of the normal simulation time course. For each time course obtained from available experimental data, we first extracted the means and standard deviations (based on the sample size information provided in each study) at each time point. Then, we divided the mean and standard deviation values by the maximal mean value in the time course for normal angiogenesis.

Next, we tested the ability of our model to capture angiogenesis impairment during delayed healing in wounds. Diabetic wounds are considered a relevant model of angiogenesis impairment because they exhibit both poor perfusion due to leaky blood vessels and a delay in healing (Okonkwo and DiPietro, 2017). Therefore, we aimed to validate our model of impaired angiogenesis by comparing our simulated VEGF, EC, and oxygen time courses (Figure 3A–C, dashed lines) with corresponding data from the wounds of diabetic mice (Figure 3A–C, dashed symbols). The diabetic-wound data sets used for these comparisons were different from the data sets that were used to develop the impaired-angiogenesis model (see the “Materials and Methods” section, Supplementary Figures S1E,F). While the model-predicted time courses for VEGF, ECs, and oxygen (Figure 3, dashed lines) during impaired angiogenesis were qualitatively similar to their respective time courses during normal angiogenesis (Figure 3, solid lines), their peak concentrations were reduced by ∼2.5-, ∼2.5-, and ∼1.7-fold, respectively, which is consistent with experimental data in the wounds of wild-type and diabetic mice (Streit et al., 2000; Ishida et al., 2004; Galeano et al., 2008; Desmet et al., 2015). In summary, our computational model captured the typical experimentally observed, injury-induced angiogenic responses during normal and delayed healing scenarios.

### Highly Influential Processes for Angiogenesis Regulation

**FIGURE 4:**
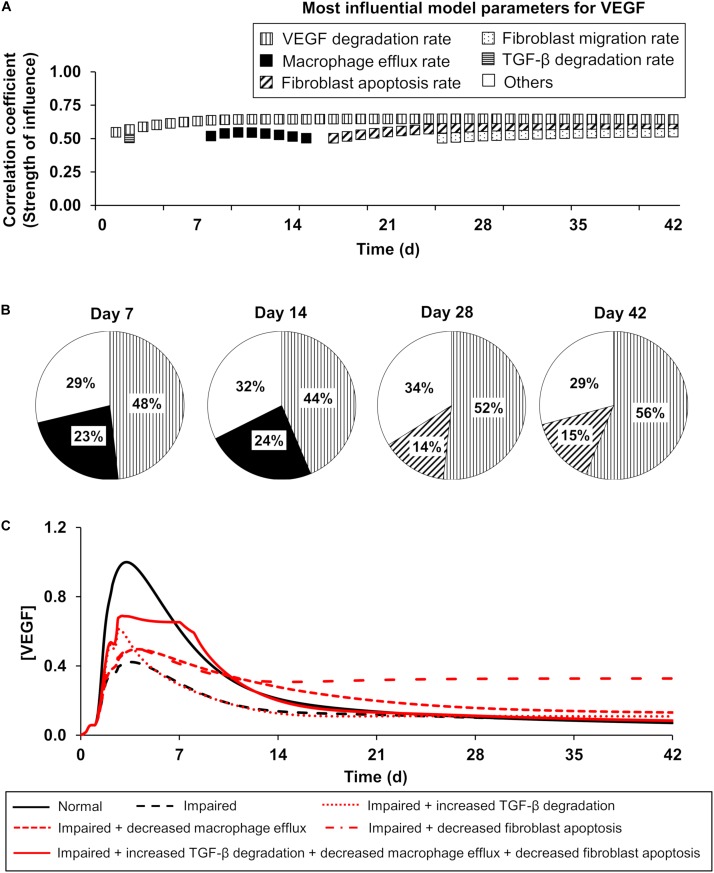
The model-predicted most influential model parameters for VEGF: temporal dependencies of the influence strength. Patterns represent a given model parameter. Horizontal stripes: TGF-β degradation rate, vertical stripes: VEGF degradation rate, solid black: macrophage efflux rate, diagonal stripes: fibroblast apoptosis rate, and dotted: fibroblast migration rate. **(A)** Patterned squares show the PRCCs (reflecting the strength of influence) between the most influential model parameters and the VEGF concentration on different days in the model-predicted time course. The model parameters whose PRCCs were above 0.5 with *P* ≤ 0.05 were identified as the most influential model parameters for a given model variable. **(B)** Pie chart shows the percentage of the 51,175 simulated wound-healing scenarios for which VEGF exhibited the *highest sensitivity* to a given parameter among the 159 model parameters. Open square (labeled as others) represents the parameters (among the 159 model parameters) to which VEGF exhibited the *highest* sensitivity in a fraction of simulations that was too small (<10%) to be considered as influential. **(C)** Model-predicted time courses of VEGF concentration during normal angiogenesis (solid black lines), impaired angiogenesis (dashed black lines), and four simulated intervention scenarios (red lines), wherein the parameters representing three of the model-identified influential processes were modified. The four interventions were simulated as follows: (1) increasing the TGF-β degradation rate by 3-fold from day 1 to day 42 post-wounding (dotted red line), (2) decreasing the macrophage efflux rate by 3-fold from day 1 to day 42 post-wounding (dashed red line), (3) decreasing the fibroblast apoptosis rate by 3-fold from day 1 to day 42 post-wounding (dash-dot red line), and (4) simultaneously increasing TGF-β degradation by 3-fold from day 1 to day 3 post-wounding, decreasing the macrophage efflux rate by 15-fold from day 2 to day 7 post-wounding, and decreasing the fibroblast apoptosis rate by 15-fold from day 1 to day 8 post-wounding (solid red line). Each model-predicted time course was normalized by dividing it by the maximal value of the normal time course of the VEGF concentration.

Given the complex nature of wound healing, several processes influence angiogenesis levels in wounds [generally reflected by the changes in the EC and VEGF levels (Altavilla et al., 2001; Kampfer et al., 2001; Hoffman et al., 2006; Okizaki et al., 2016)]. Using both the PRCC analysis and the extended LSA (see the “Materials and Methods” section), we identified the most influential processes for the EC and VEGF variables in our computational model across the simulated 42 days of inflammation, proliferation, and angiogenesis dynamics post-wounding. Based on the PRCC results, TGF-β degradation and macrophage efflux (Figure 4A, horizontally striped and solid black squares, respectively) had the strongest influence on VEGF regulation during the earlier times post-wounding (day 1 through day 14), whereas after day 16 fibroblast migration and fibroblast apoptosis had the strongest effects (Figure 4A, dotted and diagonally striped squares, respectively). Not surprisingly, the biological degradation of VEGF (Figure 4A, vertically striped square) was influential for VEGF regulation all throughout the course of wound healing. The local sensitivities calculated for the VEGF variable in our model corroborated the results of the PRCC analysis (Figure 4B). On day 7 post-wounding, VEGF was *most* sensitive to the model parameters representing the VEGF degradation rate and macrophage efflux rate in 48% and 23% of our 51,175 simulations, respectively (Figure 4B). At a later point (day 28), VEGF was *most* sensitive to the model parameters representing the VEGF degradation rate and fibroblast apoptosis rate in 52% and 14% of the 51,175 simulations, respectively (Figure 4B).

Both the PRCC analysis and the extended LSA indicated that the EC concentration was strongly influenced by VEGF degradation and macrophage efflux, particularly at earlier times (i.e., days 1 and 2) (Supplementary Figures S2A,B, solid black and dotted squares, respectively). EC migration and EC apoptosis (Supplementary Figures S2A,B, vertically and diagonally striped squares, respectively) were highly influential for EC regulation for most of the days post-wounding. Interestingly, although our computational model was developed using *in vitro* data, two of the model-identified influential processes (i.e., VEGF degradation and EC apoptosis) had been recognized in the literature as potential mechanistic drivers of impaired angiogenesis *in vivo* (Johnson and Wilgus, 2014; Treps et al., 2016). Based on these results, we modified the rates of two of these processes—namely, the EC apoptosis rate and the rate of VEGF production by macrophages—to simulate impaired angiogenesis in our model (see the section “Protein Inhibitor Modeling”).

Furthermore, we investigated whether the modulation of three other model-identified influential processes could serve as promising intervention strategies to restore VEGF levels during impaired angiogenesis. Specifically, we simulated three interventions: (1) an increase in the TGF-β degradation rate (Figure 4C, dotted red line), (2) a decrease in the macrophage efflux rate (Figure 4C, dashed red line), and (3) a decrease in the fibroblast apoptosis rate (Figure 4C, dash-dot red line). Each of these interventions induced an increase in VEGF concentration compared to its concentration during impaired angiogenesis (Figure 4C, dashed black line). The time points at which each of these interventions induced the highest increase in VEGF concentrations coincided with the time points at which the corresponding modulated processes were predicted to be influential (Figure 4A). Moreover, when we simulated an intervention by modulating all three processes at different, yet overlapping time points, the restoration of VEGF concentration was greater than that for interventions where only one process was modulated (Figure 4C, solid red line). The time points at which each process was modulated were selected with the aim of restoring the VEGF concentration to its normal angiogenesis levels (Figure 4C, solid black line). In sum, our GSA predicted six influential processes for the regulation of key angiogenic indicators (i.e., the levels of ECs and VEGF): VEGF degradation, TGF-β degradation, fibroblast migration, fibroblast apoptosis, EC migration, and EC apoptosis, as well as the optimal time points for modulating these processes.

It may be experimentally unfeasible to manipulate some of these processes—e.g., the modulation of biological degradation rates of proteins or the apoptosis rate of a given cell—during the course of wound healing. Yet, biological processes can be experimentally controlled via the addition of antibodies and inhibitors, or via the use of genetically modified animals (Altavilla et al., 2001; De Crescenzo et al., 2003; Liu et al., 2009; Stefanini et al., 2010; Wietecha and DiPietro, 2013). Therefore, as a next step, we performed a computational protein KO analysis to identify key proteins that could be experimentally targeted to regulate the model-identified influential processes.

### Protein Targets for Restoring Angiogenesis in Wounds

**FIGURE 5:**
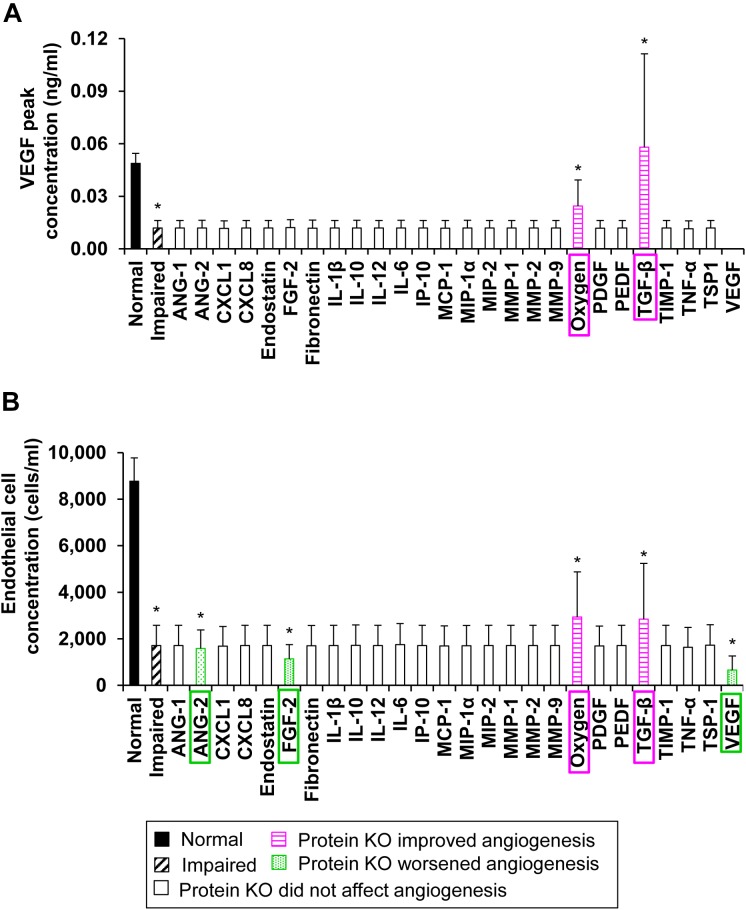
The model-predicted potential therapeutic targets: Protein KO and oxygen reduction simulation. Shown are the means and standard deviations of **(A)** VEGF and **(B)** EC peak concentrations from their simulated time courses in the “normal angiogenesis” (*N* = 205) (solid bars), “impaired angiogenesis” (*N* = 508) (dashed bars), 25 “impaired angiogenesis with protein KO” (*N* = 508 for each KO group) (open bars), and “impaired angiogenesis with oxygen reduction” (*N* = 508) (open bars) groups. Proteins whose KO increased (with *P* ≤ 0.05) the mean VEGF or mean EC peak concentrations compared to their values in the “impaired angiogenesis” group are shown in horizontally striped pink bars and the specific knocked-out protein or modified molecular species are highlighted in pink boxes. Proteins whose KO decreased (with *P* ≤ 0.05) the mean EC peak concentration compared to its mean value in the “impaired angiogenesis” group are shown in dotted green bars and the specific knocked-out proteins are highlighted in green boxes.

To identify the wound proteins (such as growth factors, cytokines, and chemokines) whose modulation would improve angiogenesis in wounds with delayed healing, we first classified our 51,175 simulations into “normal angiogenesis” and “impaired angiogenesis” (see the “Materials and Methods” section). After performing the classification, there were 205 simulations in the “normal angiogenesis” group and 538 simulations in the “impaired angiogenesis” group. Next, we simulated the KO of 25 of the 29 modeled proteins and simulated modulation of oxygen levels individually in each of the 538 simulations in the “impaired angiogenesis” group (i.e., we performed 13,988 additional simulations). Finally, we compared the mean values of the EC and VEGF peak concentrations in the “normal angiogenesis” (*N* = 205), “impaired angiogenesis” (*N* = 538), and the 26 “impaired angiogenesis with protein KO” (*N* = 538) groups of simulations. This comparison showed that the peak concentrations of ECs and VEGF were lowered (*P* ≤ 0.001) by ∼4- and ∼2.5-fold, respectively, in the “impaired angiogenesis” group compared to the “normal angiogenesis” group (Figure 5A,B, solid bars vs. dashed bars). Furthermore, the VEGF peak concentrations were increased (*P* ≤ 0.001) by ∼3- and ∼1.5-fold in the “impaired angiogenesis” group with TGF-β KO and in the “impaired angiogenesis” group with oxygen reduction, respectively (Figure 5A, horizontally striped pink bars), compared to the “impaired angiogenesis” group without any protein or oxygen modulation (Figure 5A, dashed bar). Similarly, the “impaired angiogenesis” group with the TGF-β KO and oxygen reduction increased (*P* ≤ 0.001) the EC peak concentrations (Figure 5B, horizontally striped pink bars) compared to the “impaired angiogenesis” group without any protein or oxygen modulation (Figure 5B, dashed bar) by ∼1.7- and ∼1.6-fold, respectively. At the same time, VEGF KO, FGF-2 KO, and ANG-2 KO in the “impaired angiogenesis” group decreased the EC peak concentration (Figure 5B, dotted green bars) compared to the “impaired angiogenesis” group without any protein modulation (Figure 5B, dashed bar) by ∼2.2- (*P* ≤ 0.001), ∼1.8- (*P* ≤ 0.001), and ∼1.1-fold (*P* ≤ 0.05), respectively. Based on these results, our analysis indicated that lowering the levels of TGF-β or oxygen and increasing the levels of VEGF, FGF-2, or ANG-2 may restore angiogenesis during delayed wound healing. Thus, our protein KO analysis yielded four proteins (among the 29 modeled in total) and oxygen as targets whose inhibition (TGF-β and oxygen levels) or supplementation (FGF-2, ANG-2, and VEGF) could improve angiogenesis in wounds with delayed healing.

### TGF-β Inhibitor Addition and Supplementation of FGF-2 or ANG-2 Restores Wound Angiogenesis During Delayed Healing

Our GSA, extended LSA, and protein KO analysis identified the most influential processes for angiogenesis regulation and plausible protein targets to modulate those processes. We simulated the inhibition or supplementation of three (namely, TGF-β, FGF-2, and ANG-2) of the model-identified protein targets to assess plausible intervention strategies to repair angiogenesis in wounds with delayed healing. We did not simulate any interventions involving VEGF modulation because we varied the rate of VEGF production by anti-inflammatory macrophages to simulate impaired angiogenesis in our model (see the “Materials and Methods” section). Moreover, VEGF is already a well-known pro-angiogenic protein and has been previously studied as a therapeutic agent, albeit with limited clinical success (Barrientos et al., 2014).

**FIGURE 6:**
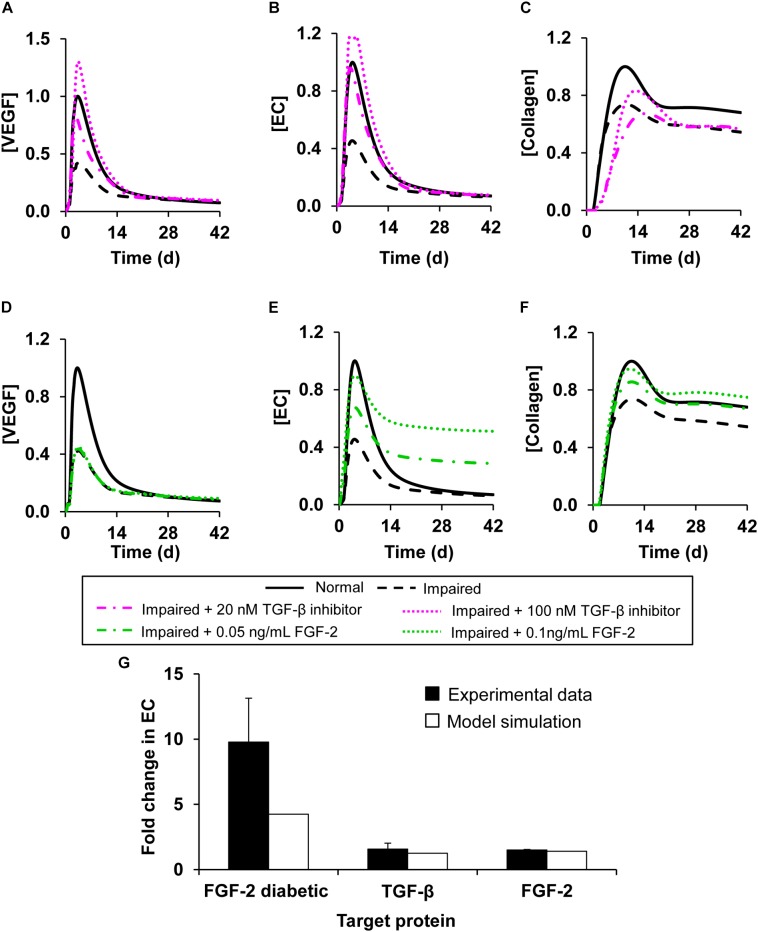
Angiogenesis regulation by modulation of model-identified proteins targets. Shown are the model-predicted **(A,D)** VEGF, **(B,E)** EC, and **(C,F)** collagen concentration time courses during normal angiogenesis (solid black lines), impaired angiogenesis (dashed black lines), and four simulated intervention scenarios, namely, **(A–C)** adding TGF-β inhibitor at concentrations of 20 nM (dash-dot pink lines) and 100 nM (dotted pink lines) and **(D–F)** supplementing FGF-2 at concentrations of 0.05ng/mL (dash-dot green lines) and 0.1 ng/mL (dotted green lines). All the interventions were introduced 1 h post-wounding. We simulated impaired angiogenesis by decreasing the VEGF production rate by anti-inflammatory macrophages by 3-fold and increasing the EC apoptosis rate by 1.2-fold. **(G)** Experimental data (solid black bars) from three separate studies shows the fold changes in EC concentrations induced by (1) addition of TGF-β inhibitor wild-type mouse wounds (*N* = 3) (Liu et al., 2009), (2) supplementation of FGF-2 in wild-type mouse wounds (*N* = 4) (Matsumoto et al., 2013), (3) and supplementation of FGF-2 in diabetic mouse wounds (*N* = 8) (Das et al., 2016). Open bars show the corresponding EC fold changes calculated in our computational model.

We first simulated interventions that involved modulating the concentration of a single protein at 1 h post-wounding. Specifically, we simulated three intervention scenarios in the impaired-angiogenesis model: supplementing a TGF-β inhibitor, supplementing FGF-2, and supplementing ANG-2. We simulated the supplementation of a TGF-β inhibitor at two different concentrations (specifically, 20 and 100 nM). The TGF-β inhibitor concentrations that we tested were arbitrarily selected based on the range (0–225 nM) used in the experimental study from which we derived the association and dissociation rate constants of the TGF-β inhibitor (De Crescenzo et al., 2003). At both concentrations, supplementing the TGF-β inhibitor led to an increase in the peak concentrations for both VEGF (Figure 6A, dash-dot and dotted pink lines) and EC (Figure 6B, dash-dot and dotted pink lines) during impaired angiogenesis. TGF-β inhibition demonstrated similar results when simulated at 24 h post-wounding (results not shown). In addition to evaluating the effect of TGF-β inhibition on VEGF and EC concentrations during impaired angiogenesis, we also evaluated the effect of this intervention on the collagen level in the wound. We sought to verify that an intervention that improves angiogenesis would not interfere with other critical processes required for successful healing of the wound (i.e., deposition of granulation tissue and wound closure). In our previous work, we have shown that TGF-β regulates collagen concentration in a wound by increasing fibroblast proliferation and by increasing fibroblast production of tropocollagen, and that lowering of TGF-β levels may lead to lower collagen levels in wounds and, subsequently, to delayed wound closure (Pakyari et al., 2013; Nagaraja et al., 2017; Stahnke et al., 2017). Indeed, TGF-β inhibition did not improve the lowered collagen levels in the impaired-angiogenesis wounds and, in fact, delayed the accumulation of collagen compared to normal healing (Figure 6C, dash-dot and dotted pink lines). Therefore, TGF-β inhibition alone was not sufficient for normalizing wound healing during impaired angiogenesis.

Next, we simulated interventions by supplementing FGF-2 (Figure 6D–F) and ANG-2 (Supplementary Figures S3A,B) at 1 h post-wounding in the impaired-angiogenesis model. Supplementation of FGF-2 at concentrations of 0.05 and 0.1 ng/mL led to an increase in the peak concentrations of ECs during impaired angiogenesis (Figure 6E, dash-dot and dotted green lines). Supplementation of ANG-2 at concentrations of 0.05 and 0.015 ng/mL yielded a response similar to FGF-2 supplementation (Supplementary Figure S3B, dash-dot and dotted orange lines). However, supplementation of either FGF-2 (Figure 6D, dash-dot and dotted green lines) or ANG-2 (Supplementary Figure S3A, dash-dot and dotted orange lines) did not induce any change in the VEGF concentration during impaired angiogenesis. Interestingly, supplementation of either FGF-2 or ANG-2 alone did indeed lead to an increase in the collagen level during impaired angiogenesis (Figure 6F, dash-dot and dotted green lines, Supplementary Figure S3C dash-dot and dotted orange line). Similarly to TGF-β inhibition, the response to the interventions with FGF-2 and ANG-2 supplementations did not change when they were simulated at 24 h post-wounding (results not shown). To validate our predictions regarding the interventions with single protein modulation, we compared the model-predicted fold changes in the EC concentration after the simulated interventions during normal and impaired angiogenesis with the corresponding fold changes calculated from available experimental data (Figure 6G, open bars vs. solid bars). From the data, we calculated the EC fold changes in the wounds of wild-type and diabetic mice induced by the supplementation of FGF-2 (Matsumoto et al., 2013; Das et al., 2016), and the EC fold changes in the wounds of wild-type mice induced by the supplementation of a TGF-β inhibitor (Liu et al., 2009). In all our comparisons, the model predictions showed reasonable agreement with the experimental data (Figure 6G).

**FIGURE 7:**
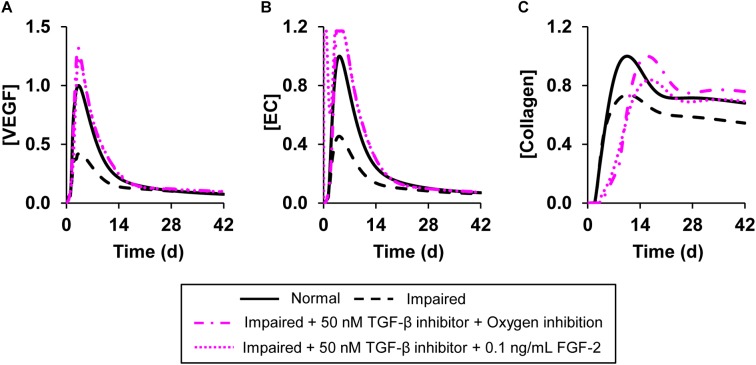
Angiogenesis regulation by simultaneous modulation of multiple model-identified proteins targets. Shown are the model-predicted **(A)** VEGF, **(B)** EC, and **(C)** collagen concentration time courses during normal angiogenesis (solid black lines), impaired angiogenesis (dashed black lines), and two simulated intervention scenarios wherein multiple proteins and oxygen were simultaneously modulated. In the first intervention, we simulated the simultaneous addition of TGF-β inhibitor at a concentration of 50 nM and decreased the wound oxygen levels to half of normal oxygen levels (dash-dot pink lines). In the second intervention, we simulated the simultaneous addition of TGF-β inhibitor at a concentration of 50 nM along with supplementation of FGF-2 at a concentration of 0.1 ng/mL (dotted pink lines). We simulated impaired angiogenesis by decreasing the VEGF production rate by anti-inflammatory macrophages by 3-fold and increasing the EC apoptosis rate by 1.2-fold. All the interventions were introduced 1 h post-wounding except the reduction in wound oxygen levels which was simulated at day 0 (i.e., at the time of injury).

Our simulations of interventions, each of which targeted only a single protein, indicated that the individual proteins we modified successfully normalized either the VEGF and EC concentrations (indicators of angiogenesis) or the collagen level (indicator of wound closure). Therefore, we simulated interventions that involved modulation of the levels of two proteins simultaneously or modulating the levels of one protein and oxygen simultaneously. Specifically, we simulated three interventions: (1) supplementing a TGF-β inhibitor with FGF-2 (Figure 7, dotted pink lines), (2) supplementing a TGF-β inhibitor and reducing oxygen levels in the wound (Figure 7, dash-dot pink lines), and (3) supplementing a TGF-β inhibitor with ANG-2 (Supplementary Figure S3, solid pink lines) in the impaired-angiogenesis model. For simulating oxygen reduction we introduced a parameter that modulated the amount of oxygen released from newly formed blood vessels at the wound site as previously described (Schugart et al., 2008). We used this approach because we cannot simulate the removal of a finite concentration of a given protein or oxygen. Moreover, unlike we did for TGF-β, oxygen activity cannot be modulated by inhibitor binding. All three interventions led to an increase in the peak concentration of VEGF (Figure 7A and Supplementary Figure S3A), ECs (Figure 7B and Supplementary Figure S3B), and collagen (Figure 7C and Supplementary Figure S3C). However, there were small, yet noticeable differences in the EC time course between the intervention with only TGF-β inhibition (Figure 6B) and the intervention with TGF-β inhibition combined with oxygen reduction. In the former case, the EC concentration peaked around day 5 post-wounding (Figure 6B, pink dotted line), whereas when supplementation of a TGF-β inhibitor was combined with a lowering of oxygen levels, the EC concentration had a second earlier peak around day 1 post-wounding (Figure 7B, pink dotted line). In our model, we defined a feedback function that increases the rate of VEGF production by pro-inflammatory macrophages when the oxygen concentration is lowered. This feedback led to increased VEGF levels at the beginning of wound healing. This, is turn, increased EC chemotaxis, which resulted in the second peak in the EC concentration. In sum, our analysis showed that TGF-β, FGF-2, and ANG-2 could be potential molecular targets whose combined modulation, together with wound hypoxia, may restore angiogenesis and thereby accelerate closure in wounds with delayed healing.

## Discussion

Balanced angiogenesis is essential for a normal wound-healing response. Strategies for regulating angiogenesis in wounds are limited owing to the number and complexity of the molecular and cellular processes constituting angiogenesis. Our computational model of wound healing successfully captured the time courses of platelets, nine cell types, 29 proteins, and oxygen in a wound during normal and impaired angiogenesis, which was validated by comparisons with experimental data (Figure 2, 3). Of the 133 processes represented in the model, we identified six (i.e., VEGF degradation, fibroblast migration, fibroblast apoptosis, TGF-β degradation, EC migration, and EC apoptosis) as the most influential processes affecting the regulation of angiogenesis in wounds (Figure 4). Moreover, among the 30 modeled molecular species (wound proteins and oxygen), we identified four proteins (i.e., VEGF, TGF-β, FGF-2, and ANG-2) and oxygen as potential therapeutic targets whose modulation may stimulate wound angiogenesis (Figure 5). Finally, we predicted that intervention strategies involving simultaneous targeting of either two proteins or one protein and the wound oxygen level may increase angiogenesis more efficiently than strategies that target either a single protein or the wound oxygen level alone (Figure 6, 7 and Supplementary Figure S3).

Regeneration of functional blood vessels is crucial for the transport of oxygen and nutrients to the wound tissue, which contains many metabolically active immune cells, such as neutrophils, macrophages, and fibroblasts (Yoo and Kwon, 2013; Okonkwo and DiPietro, 2017). Given the contribution of angiogenesis to the normal functioning of cells from different phases of wound healing (i.e., inflammation and proliferation), its dysregulation at any given time can give rise to a variety of wound pathologies, such as chronicity (characterized by impaired angiogenesis) or fibrosis (characterized by excessive angiogenesis) (Darby and Hewitson, 2016; Sorg et al., 2017). Here, we focused on pathologies characterized by impaired angiogenesis, such as chronic wounds and wounds with delayed healing. Chronic wounds (e.g., diabetic wounds and ulcers) are among the leading causes of lower-extremity amputations, and present a major burden for both patients and clinicians (Tang et al., 2013). Consequently, therapies for restoring angiogenesis are constantly being investigated and improved. Current therapies include hyperbaric oxygen therapy, tissue-engineered dressings, and growth-factor-based therapies (such as topical application of platelet-derived growth factor) (Wietecha and DiPietro, 2013; Ma et al., 2016; Okonkwo and DiPietro, 2017; Sorg et al., 2017). Although these therapies have shown moderate improvement of angiogenesis in experimental settings, none have proven to be efficacious in clinical trials (Wietecha and DiPietro, 2013; Logsdon et al., 2014). Ongoing efforts are directed toward both the improvement of existing therapies (Gagliardi, 2016; Ho et al., 2017; Kaplani et al., 2018) and identification of new therapies. The limited translational potential (from animal models to the clinic) of existing therapies can be attributed to a lack of mechanistic understanding of how they work *in vivo*. Although our computational model is built using *in vitro* experimental data characterizing the various cellular and molecular signaling processes involved in angiogenesis, it realistically captures both normal and impaired angiogenesis *in vivo* (Figure 2, 3). In this way, the model may be useful for ongoing investigations aimed at identifying new mechanism-based therapeutic interventions for impaired angiogenesis, which might have better translational capabilities.

It is generally accepted that the abundance of ECs, as well as the VEGF levels, reflect the angiogenic activity in wounds (Altavilla et al., 2001; Kampfer et al., 2001; Okizaki et al., 2016). Given the numerous cell types and proteins that participate in angiogenesis, EC and VEGF activities may be regulated by several mechanistic factors at different times during the wound-healing response. A computational approach makes it possible to systematically explore such therapeutic targets and the optimal time points to modulate them. For example, computational modeling of the VEGF/VEGFR pathway has allowed for the identification of interventions targeting different molecular species in that pathway (Stefanini et al., 2010; Finley et al., 2011). Furthermore, multiple groups have developed computational models describing distinct components of the angiogenesis process (e.g., blood-vessel density changes in response to macrophage-derived angiogenesis factors, the distribution of VEGF in skeletal muscle, and the sprouting/elongation of new blood vessels) (Logsdon et al., 2014). However, because angiogenesis does not occur in isolated steps, a normal angiogenesis response will depend on the normal signaling of cells and proteins in other wound-healing phases (i.e., inflammation and proliferation) and vice versa. In accord with this view, our model describes not only the kinetics of angiogenic cells and proteins (such as ECs, blood vessels, and eight angiogenic proteins), but also those of 12 inflammatory proteins, 11 proliferative proteins, 4 inflammatory and 3 proliferative cell types, and their interactions with angiogenic cells and proteins. Indeed, our modeling results showed that the concentrations of both ECs and VEGF are influenced by distinct molecular signaling processes at different times during wound healing (Figure 4). Interestingly, of the four model-identified angiogenesis-influencing processes, three of them, namely TGF-β degradation, fibroblast migration, and apoptosis, mainly govern the *proliferative* phase of the wound-healing response (Serra et al., 2017; Kaplani et al., 2018) while macrophage efflux mainly governs its *inflammatory* phase. Thus, computational models that reliably capture the entire angiogenesis process in the proper mechanistic context (i.e., the dynamic involvement of angiogenesis in other wound-healing phases) can guide the exploration of new therapeutic interventions in three ways: (1) by widening the pool of potential therapeutic targets, (2) by accounting for redundancies in the function of different proteins, and (3) by revealing any undesirable effects that angiogenesis-restoring strategies may have on other wound-healing outcomes (e.g., wound closure).

Wound proteins are among the most appealing potential targets for treating a wide variety of wound pathologies, including impaired angiogenesis (Bodnar, 2015; Okonkwo and DiPietro, 2017; Serra et al., 2017; Sorg et al., 2017). Interestingly, three of the model-identified targets [namely, FGF-2, VEGF, and TGF-β (Figure 5)] have previously been investigated as therapeutic targets to improve angiogenesis in wounds. Topical or oral administration of bFGF and VEGF has been investigated as a therapy for improving the healing of diabetic ulcers and wounds (Galiano et al., 2004; Matsumoto et al., 2013; Das et al., 2016). Furthermore, TGF-β inhibitors have been shown to work synergistically with VEGF to improve capillary growth in mouse wounds (Ferrari et al., 2009; Liu et al., 2009; Jing et al., 2015). In addition to proteins, molecular species, such as oxygen, affect the angiogenic activity in wounds. Acute hypoxia present during the initial stages of wound healing induces the release of hypoxia inducible factor-1 (HIF-1), which promotes angiogenesis by stimulating the production of VEGF by inflammatory cells and ECs (Jing et al., 2015). In contrast, current therapies for non-healing wounds involve providing exogenous oxygen to wounds (i.e., inducing hyperoxia) (Hopf et al., 2005). Yet, there is an optimal level of oxygen beyond which the beneficial effects of hyperoxia diminish (Schugart et al., 2008). In accord with these notions, our model simulations showed that interventions involving the lowering of the oxygen level restored angiogenesis in wounds up to a certain limit. The efficacy of these interventions increased when they were combined with supplementation of other pro-angiogenic factors during hypoxia (Figure 6). However, despite their success in pre-clinical animal models, therapies with VEGF, FGF-2, and HIF-1 as therapeutic agents have failed to increase angiogenesis in clinical trials (Hanft et al., 2008; Creager et al., 2011; Logsdon et al., 2014).

The common feature in all of these interventions is that they target a single protein or a single signaling pathway (i.e., VEGF, FGF-2, TGF-β, or HIF-1). However, many wound proteins perform different functions depending on the stage of wound repair. For example, TGF-β modulates angiogenesis during wound healing by regulating EC proliferation and migration (Ramsauer and D’Amore, 2007; Ferrari et al., 2009). It also promotes collagen deposition and accelerates wound closure during the proliferative phase of wound healing (Pakyari et al., 2013). Indeed, our modeling results showed that, although TGF-β inhibition improved angiogenesis (by increasing the levels of VEGF and ECs), it did not improve collagen deposition or wound closure in delayed-healing wounds (Figure 6A–C). Yet, when TGF-β inhibition was combined with supplementation of FGF-2 or ANG-2, both angiogenesis and collagen deposition were restored to levels observed during a normal healing response (Figure 7C). Interestingly, recent efforts—as well as our model simulations—have highlighted the advantages of a combinatorial approach (i.e., simultaneously targeting multiple proteins/molecules) to achieve higher efficacies in restoring angiogenesis (Allen et al., 2014; Gao et al., 2015). In fact, interventions involving the combined supplementation of VEGF with other angiogenic proteins, such as ANG-1, FGF-2, and TSP-1, have been investigated for different conditions in which angiogenesis is impaired (Gerber et al., 1998; Arsic et al., 2003; Liu et al., 2009). With routine discoveries of new targets for angiogenesis regulation [e.g., the chemokines CCL2 and CCL5 and their receptors (Bodnar, 2015; Ridiandries et al., 2017)], an important consideration for future studies investigating potential interventions to restore angiogenesis is the evaluation of possible compensatory metabolic routes activated by the blockade/supplementation of the therapeutic target(s) being investigated. The efficacy and efficiency of such investigations can be increased by the use of computational modeling approaches, which can systematically and rapidly assess the plausible outcomes of multifactorial treatments in advance of the laborious experimental testing.

Our computational model provides opportunities for different types of predictive analysis and hypothesis generation. To demonstrate this capability, we used the model to perform simulations and generate two new insights. First, we used the model to generate therapeutic interventions that highlighted the importance of the interaction between angiogenesis and other wound-healing phases, which is frequently overlooked in therapeutic investigations. Our predictions showed that, in addition to modulating EC dynamics and the levels of key proteins such as VEGF, angiogenesis could be promoted by the timely modulation of *inflammatory* and *proliferative* processes. We showed that the combined modulation of macrophage efflux, TGF-β degradation, and fibroblast apoptosis within an optimal range of time points post-wounding (days 2–7, days 1–3, and days 1–8, respectively) could increase VEGF levels during impaired angiogenesis (Figure 4C). Second, we used the model to identify a potential therapeutic role for ANG-2 in wound healing. ANG-2 is a known angiogenic protein and has previously been investigated as a therapeutic target for different types of cancer (typically characterized by heightened angiogenesis). Although inhibition of ANG-2 has been shown to reduce angiogenesis (Comunanza and Bussolino, 2017; Zirlik and Duyster, 2018), surprisingly, ANG-2 supplementation has not been investigated as a therapy for increasing angiogenesis in delayed-healing wounds. Our model-predicted interventions could guide future investigations for restoring angiogenesis in wounds-specifically, those involving the supplementation of ANG-2 alone or in combination with TGF-β inhibitors (Supplementary Figures S3B,C).

Our computational model has a number of limitations, owing to the assumptions made during model development, which were necessary to capture the complex nature of angiogenesis. First, we did not explicitly model hypoxia-induced HIF-1 signaling, which is known to initiate angiogenesis (Jing et al., 2015). However, we captured the role of hypoxia in angiogenesis regulation by using a feedback function [*f_V EGF_*(*O*) in Supplementary Table S2] that changes the rate of VEGF production based on the oxygen levels in the wound (van Vlimmeren et al., 2010). Second, angiogenesis is a complex, multi-step process, which we did not model in full detail. To ascertain modeling feasibility, we used a number of simplifying assumptions and empirically derived feedback functions to reduce the model complexity of the angiogenesis process while retaining its key features. Finally, angiogenesis impairment may be affected by a variety of factors, such as wound infection, disease (e.g., diabetes and atherosclerosis), and wound origin (e.g., burn wounds and ulcers) (Hoffman et al., 2006; Ling et al., 2012; Okonkwo and DiPietro, 2017). Because our work is focused primarily on injuries, and mechanistic data regarding how such factors contribute to angiogenesis are lacking, we do not account for such additional layers of complexity in our model representation of angiogenesis.

Computational models offer a systematic way to study complex biological systems. In addition to providing mechanistic insights, they can act as surrogate systems to design, test, and optimize new therapies for different pathological conditions (Logsdon et al., 2014; Flegg et al., 2015). Our modeling results illustrate the utility of a computational approach for investigating therapies to restore angiogenesis in wounds. Rigorous experimental testing of our modeling results may contribute to the identification of new therapies for restoring angiogenesis in wounds with delayed healing.

The computational model developed in this work can further be used for different research purposes. For example, one could use the model, in combination with our recently published analysis methodology, to identify diagnostic or prognostic molecular indicators (i.e., potential biomarkers) of wound-healing pathologies, such as impaired angiogenesis and chronic inflammation (Nagaraja et al., 2018). Using the model, one may predictively assess the effects of newly conceived therapeutic interventions on multiple wound-healing characteristics, such as the levels of ECs, collagen, and wound oxygen. Moreover, our model can be extended to study other aspects of wound healing and immunity in general, such as wound infection dynamics, the influence of intracellular signaling on overall inflammation kinetics (Tomaiuolo et al., 2016), and the role of adaptive immunity in wound healing. Finally, our model can be combined with mechanics-based computational models of skin wounds (Zhao et al., 2017) to investigate wound healing as a spatiotemporal process.

## Software Availability

The MATLAB code for our computational analyses is freely available and can be downloaded from GitHub (https://github.com/BHSAI/Angiogenesis).

## Author Contributions

SN, AYM, and JR conceptualized the research performed in this study. SN performed the computational analysis and comparisons with experimental and clinical data. SN, AYM, JR, LAD, and LC wrote and edited the manuscript. All authors read and approved the final manuscript.

## Disclaimer

The opinions and assertions contained herein are private views of the authors and are not to be construed as official or as reflecting the views of the United States Army, United States Department of Defense, or The Henry M. Jackson Foundation for the Advancement of Military Medicine, Inc. This article has been approved for public release with unlimited distribution.

## Conflict of Interest Statement

The authors declare that the research was conducted in the absence of any commercial or financial relationships that could be construed as a potential conflict of interest.
