# Supplementary material for: Predictive Approach Identifies Molecular Targets and Interventions to Restore Angiogenesis in Wounds With Delayed Healing
Source: Front Physiol. 2019 May 28;10:636. doi: 10.3389/fphys.2019.00636 (PMC6547939; doi:10.3389/fphys.2019.00636)
Supplement: Supplementary file 1 [file Data_Sheet_1.pdf]

SUPPLEMENTARY MATERIAL

Predictive approach identifies molecular targets and interventions to restore angiogenesis in wounds with delayed healing

by Sridevi Nagaraja, Lin Chen, Luisa A. DiPietro, Jaques Reifman, and
Alexander Y. Mitrophanov

Table S1. List of the biological processes included in the model.

Process #	Description of the biological process
1	Platelet degradation
2	TGF-β release by platelets
3	Neutrophil chemotaxis
4	Neutrophil apoptosis
5	Apoptotic neutrophil phagocytosis by pro-inflammatory macrophages
6	Pro-inflammatory macrophage chemotaxis
7	Macrophage phenotype conversion
8	Macrophage efflux by lymphatic system
9	TGF-β production by pro-inflammatory macrophages
10	TGF-β production by anti-inflammatory macrophages
11	TGF-β degradation
12	PDGF production by pro-inflammatory macrophages
13	PDGF production by anti-inflammatory macrophages (10% of k_{PDGF_pro})
14	PDGF degradation
15	TNF-α production by pro-inflammatory macrophages
16	TNF-α production by anti-inflammatory macrophages
17	TNF-α degradation
18	IL-1β production by pro-inflammatory macrophages
19	IL-1β production by anti-inflammatory macrophages
20	IL-1β degradation
21	IL-6 production by pro-inflammatory macrophages
22	IL-6 production by anti-inflammatory macrophages (10% of $k_{IL\text{-}6pro}$)
23	IL-6 degradation
24	IL-10 production by pro-inflammatory macrophages
25	IL-10 production by anti-inflammatory macrophages
26	IL-10 degradation
27	CXCL8 production by pro-inflammatory macrophages
28	CXCL8 production by anti-inflammatory macrophages
29	CXCL8 degradation
30	IL-12 production by pro-inflammatory macrophages
31	IL-12 degradation
32	MIP-1α production by pro-inflammatory macrophages
33	MIP-1α production by anti-inflammatory macrophages
34	MIP-1α degradation
35	MIP-2 production by pro-inflammatory macrophages
36	MIP-2 production by anti-inflammatory macrophages
37	MIP-2 degradation
38	IP-10 production by pro-inflammatory macrophages
39	IP-10 production by anti-inflammatory macrophages
40	IP-10 degradation
41	FGF-2 production by anti-inflammatory macrophages
42	TNF-α production by active neutrophils
43	IL-1β production by active neutrophils

44	IL-6 production by active neutrophils
45	Inhibition of TNF-α by IL-10
46	Inhibition of IL-6 by IL-10
47	Inhibition of IL-1β by IL-10
48	Fibroblast chemotaxis
49	Fibroblast proliferation
50	Fibroblast apoptosis
51	Inhibition of TNF-α by TGF-β
52	Inhibition of IL-1β by TGF-β
53	Inhibition of IL-1β by IL-6
54	Inhibition of TNF-α by IL-6
55	Inhibition of IL-12 by TNF-α
56	Upregulation of IL-6 by TGF-β
57	Upregulation of IL-10 by TGF-β
58	Myofibroblast apoptosis
59	Fibronectin production by pro-inflammatory macrophages
60	Fibronectin production by fibroblasts
61	Fibronectin degradation
62	FGF-2 production by pro-inflammatory macrophages
63	FGF-2 degradation
64	MMP-9 production by pro-inflammatory macrophages
65	MMP-9 production by fibroblasts
66	MMP-9 degradation
67	MMP-1 production by pro-inflammatory macrophages
68	MMP-1 degradation
69	MMP-2 production by pro-inflammatory macrophages
70	MMP-2 degradation
71	TIMP-1 production by fibroblasts
72	TIMP-1 degradation
73	IL-10 production by fibroblasts
74	CXCL8 production by fibroblasts
75	Tropocollagen production by fibroblasts
76	Collagen degradation due to MMPs
77	Collagen degradation due to fibroblast enzymes
78	TGF-β production by fibroblasts
79	Tropocollagen polymerization into intermediate collagen
80	Intermediate collagen polymerization into collagen fiber
81	IL-6 production by fibroblasts
82	TIMP-1 production by pro-inflammatory macrophages
83	MCP-1 production by pro-inflammatory macrophages
84	MCP-1 degradation
85	Myofibroblast proliferation
86	Upregulation of tropocollagen production by MCP-1
87	VEGF production by pro-inflammatory macrophages
88	VEGF production by anti-inflammatory macrophages

89	VEGF production by fibroblasts
90	VEGF degradation
91	ANG-1 production by endothelial cells
92	ANG-1 production by fibroblasts
93	ANG-1 degradation
94	ANG-2 production by endothelial cells
95	ANG-2 production by fibroblasts
96	ANG-2 degradation
97	TSP-1 production by pro-inflammatory macrophages
98	TSP-1 production by fibroblasts
99	TSP-1 production by endothelial cells
100	TSP-1 degradation
101	Endostatin production by endothelial cells
102	Endostatin degradation
103	Capillary growth
104	Capillary tip-tip anastomosis
105	Capillary tip-sprout anastomosis
106	Capillary remodeling
107	Oxygen release from blood vessels
108	Oxygen degradation
109	Oxygen consumption by macrophages
110	Oxygen consumption by fibroblasts
111	Endothelial cell proliferation
112	Endothelial cell apoptosis
113	Upregulation of ANG-2 production by VEGF
114	CXCL8 production by endothelial cells
115	Endothelial cell chemotaxis
116	EC tips anastomosing to form blood vessel sprouts
117	Differentiation of blood vessel tip
118	IL-6 production by endothelial cells
119	VEGF production by endothelial cells
120	Endothelial cell apoptosis in blood vessels
121	PEDF production by pro-inflammatory macrophages
122	PEDF production by endothelial cells
123	PEDF degradation
124	Keratinocyte chemotaxis
125	Keratinocyte proliferation
126	Production of KGF by fibroblasts
127	KGF degradation
128	CXCL1 production by pro-inflammatory macrophages
129	CXCL1 degradation
130	VEGF production by keratinocytes
131	CXCL1 production by fibroblasts
132	Oxygen level control
133	Fractional contribution of TGF-β and PDGF to fibroblast chemotaxis

Table S2. List of model parameters with their assigned numbers (P#), the process number they represent, descriptions of their function, values with respective units, feedback functions, model variables, model equations, and the corresponding literature references.

P#	Proc-ess#	Model parameter description	Value	Unit	Reference
1	1	Platelet degradation rate	0.69	h^{-1}	(1)
2	2	Rate of TGF-β release by platelets	1.25×10^{-8}	$ng \cdot P^{-1} \cdot h^{-1}$	(2, 3)
3	3	Chemotactic migration of neutrophils to the wound site (TGF-β-dependent)	300	mL^{-1}	Assumed§
4	4	Rate of neutrophil apoptosis	0.1	h^{-1}	(4)
5	5	Rate of apoptotic neutrophil phagocytosis by pro-inflammatory macrophages	2.00	$N \cdot M^{-1} \cdot h^{-1}$	(5)
6	5	Apoptotic neutrophil phagocytosis parameter	4.71×10^{6}	cells/mL	(5)
7	6	Chemotactic migration of pro-inflammatory macrophages to the wound site (TGF-β-dependent)	400	mL^{-1}	Assumed§
8	7	Rate of macrophage phenotype conversion	0.1	h^{-1}	(5)
9	7	Macrophage phenotype conversion parameter	1.0×10^{6}	cells/mL	(5)
10	8	Rate of macrophage efflux by lymphatic system	8.30×10^{-3}	h^{-1}	(6)
11	9	Rate of TGF-β production by pro-inflammatory macrophages	1.88×10^{-6}	$ng \cdot cell^{-1} \cdot h^{-1}$	(7)
12	10	Rate of TGF-β production by anti-inflammatory macrophages	1.60×10^{-8}	$ng \cdot cell^{-1} \cdot h^{-1}$	(8)
13	11	TGF-β degradation rate	0.693	h^{-1}	(9)
14	12	Rate of PDGF production by pro-inflammatory macrophages	6.00×10^{-8}	$ng \cdot cell^{-1} \cdot h^{-1}$	(10)
15	13	Rate of PDGF production by anti-inflammatory macrophages (10% of k_{PDGF_pro})	6.00×10^{-9}	$ng \cdot cell^{-1} \cdot h^{-1}$	Assumed*
16	14	PDGF degradation rate	0.173	h^{-1}	(11)
17	15	Rate of TNF-α production by pro-inflammatory macrophages	3.46×10^{-7}	$ng \cdot cell^{-1} \cdot h^{-1}$	(12)
18	16	Rate of TNF-α production by anti-inflammatory macrophages	4.29×10^{-8}	$ng \cdot cell^{-1} \cdot h^{-1}$	(12)
19	17	TNF-α degradation rate	0.5331	h^{-1}	(13)
20	18	Rate of IL-1β production by pro-inflammatory macrophages	1.23×10^{-6}	$ng \cdot cell^{-1} \cdot h^{-1}$	(12)
21	19	Rate of IL-1β production by anti-inflammatory macrophages	2.45×10^{-7}	$ng \cdot cell^{-1} \cdot h^{-1}$	(12)
22	20	IL-1β degradation rate	0.1732	h^{-1}	(14)
23	21	Rate of IL-6 production by pro-inflammatory macrophages	1.18×10^{-6}	$ng \cdot cell^{-1} \cdot h^{-1}$	(15)
24	22	Rate of IL-6 production by anti-inflammatory macrophages (10% of $k_{IL\text{-}6pro}$)	1.18×10^{-7}	$ng \cdot cell^{-1} \cdot h^{-1}$	Assumed*
25	23	IL-6 degradation rate	0.462	h^{-1}	(13)
26	24	Rate of IL-10 production by pro-inflammatory macrophages	7.60×10^{-8}	$ng \cdot cell^{-1} \cdot h^{-1}$	(12)
27	25	Rate of IL-10 production by anti-inflammatory macrophages	1.55×10^{-7}	$ng \cdot cell^{-1} \cdot h^{-1}$	(12)
28	26	IL-10 degradation rate	0.193	h^{-1}	(4)
29	27	Rate of CXCL8 production by pro-inflammatory macrophages	2.50×10^{-6}	$ng \cdot cell^{-1} \cdot h^{-1}$	(16)
30	28	Rate of CXCL8 production by anti-inflammatory macrophages	6.94×10^{-7}	$ng \cdot cell^{-1} \cdot h^{-1}$	(8)
31	29	CXCL8 degradation rate	0.693	h^{-1}	(17)
32	30	Rate of IL-12 production by pro-inflammatory macrophages	8.33×10^{-7}	$ng \cdot cell^{-1} \cdot h^{-1}$	(16)
33	31	IL-12 degradation rate	0.05775	h^{-1}	(18)
34	32	Rate of MIP-1α production by pro-inflammatory macrophages	3.61×10^{-8}	$ng \cdot cell^{-1} \cdot h^{-1}$	(19)
35	33	Rate of MIP-1α production by anti-inflammatory macrophages	4.25×10^{-8}	$ng \cdot cell^{-1} \cdot h^{-1}$	(19)
36	34	MIP-1α degradation rate	0.385	h^{-1}	(17)
37	35	Rate of MIP-2 production by pro-inflammatory macrophages	1.84×10^{-7}	$ng \cdot cell^{-1} \cdot h^{-1}$	(19)
38	36	Rate of MIP-2 production by anti-inflammatory macrophages	1.92×10^{-7}	$ng \cdot cell^{-1} \cdot h^{-1}$	(19)
39	37	MIP-2 degradation rate	0.2772	h^{-1}	(20)
40	38	Rate of IP-10 production by pro-inflammatory macrophages	7.50×10^{-7}	$ng \cdot cell^{-1} \cdot h^{-1}$	(16)
41	39	Rate of IP-10 production by anti-inflammatory macrophages	1.56×10^{-6}	$ng \cdot cell^{-1} \cdot h^{-1}$	(16)
42	40	IP-10 degradation rate	0.1732	h^{-1}	(21)

43	41	Rate of FGF-2 production by anti-inflammatory macrophages	1.87×10^{-7}	$ng \cdot cell^{-1} \cdot h^{-1}$	(22)
44	42	Rate of TNF-α production by active neutrophils	1.66×10^{-8}	$ng \cdot cell^{-1} \cdot h^{-1}$	(7)
45	43	Rate of IL-1β production by active neutrophils	1.70×10^{-8}	$ng \cdot cell^{-1} \cdot h^{-1}$	(7)
46	44	Rate of IL-6 production by active neutrophils	8.30×10^{-10}	$ng \cdot cell^{-1} \cdot h^{-1}$	(7)
47-49	45	Parameters of the feedback function f_1 describing the inhibition of TNF-α by IL-10: $f_1 = P_{47} e^{P_{48} IL\text{-}10} + P_{49}$	$P_{47} = 0.4666$<br>$P_{48} = -1.528$<br>$P_{49} = 0.5332$		(23)
50-52	46	Parameters of the feedback function f_2 describing the inhibition of IL-6 by IL-10: $f_2 = P_{50} e^{P_{51} IL\text{-}10} + P_{52}$	$P_{50} = 0.3298$<br>$P_{51} = -1.189$<br>$P_{52} = 0.6695$		(23)
53-55	47	Parameters of the feedback function f_3 describing the inhibition of IL-1β by IL-10: $f_3 = P_{53} e^{P_{54} IL\text{-}10} + P_{55}$	$P_{53} = 0.6334$<br>$P_{54} = -1.794$<br>$P_{55} = 0.3667$		(23)
56	48	Fibroblast influx rate	100	mL^{-1}	Assumed[§]
57	49	Fibroblast proliferation rate	3.85×10^{-2}	h^{-1}	(24)
58	50	Fibroblast apoptosis rate	3.75×10^{-2}	h^{-1}	(25)
59-61	51	Parameters of the feedback function f_4 describing the inhibition of TNF-α by TGF-β: $f_4 = P_{59} e^{P_{60} TGF\text{-}\beta} + P_{61}$	$P_{59} = 0.6211$<br>$P_{60} = -0.8305$<br>$P_{61} = 0.4466$		(26)
62-64	52	Parameters of the feedback function f_5 describing the inhibition of IL-1β by TGF-β: $f_5 = P_{62} e^{P_{63} TGF\text{-}\beta} + P_{64}$	$P_{62} = 0.69$<br>$P_{63} = -20.37$<br>$P_{64} = 0.31$		(26)
65-66	53	Parameters of the feedback function f_6 describing the inhibition of IL-1β by IL-6: $f_6 = \frac{P_{65}}{(P_{65} + IL\text{-}6^{P_{66}})}$	$P_{65} = 4.459$<br>$P_{66} = 0.1571$		(27)
67-68	54	Parameters of the feedback function f_7 describing the inhibition of TNF-α by IL-6: $f_7 = \frac{P_{67}}{(P_{67} + IL\text{-}6^{P_{68}})}$	$P_{67} = 4.488$<br>$P_{68} = 0.1541$		(27)
69-71	55	Parameters of the feedback function f_8 describing the inhibition of IL-12 by TNF-α: $f_8 = P_{69} e^{P_{70} TNF\text{-}\alpha} + P_{71}$	$P_{69} = 0.8671$<br>$P_{70} = -2.794$<br>$P_{71} = 0.1307$		(28)
72	56	Parameter of the feedback function f_9 describing the upregulation of IL-6 by TGF-β: $f_9 = \frac{P_{72} TGF\text{-}\beta}{(1 + TGF\text{-}\beta)}$	0.9821		(29)
73	57	Parameter of the feedback function f_{10} describing the upregulation of IL-10 by TGF-β: $f_{10} = \frac{P_{73} TGF\text{-}\beta}{(1 + P_{73} TGF\text{-}\beta)}$	274.5		(30)
74	58	Myofibroblast apoptosis rate	0.02	h^{-1}	(31)
75	59	Rate of fibronectin production by pro-inflammatory macrophages	1.0×10^{-5}	$ng \cdot cell^{-1} \cdot h^{-1}$	(32)
76	60	Rate of fibronectin production by fibroblasts	9.65×10^{-5}	$ng \cdot cell^{-1} \cdot h^{-1}$	(33)
77	61	Fibronectin degradation rate	0.0330	h^{-1}	(34)
78	62	Rate of bFGF production by pro-inflammatory macrophages	1.3×10^{-7}	$ng \cdot cell^{-1} \cdot h^{-1}$	(22)
79	63	FGF-2 degradation rate	0.0912	h^{-1}	(35)
80	64	Rate of MMP-9 production by pro-inflammatory macrophages	1.3×10^{-3}	$ng \cdot cell^{-1} \cdot h^{-1}$	(36)
81	65	Rate of MMP-9 production by fibroblasts	9.03×10^{-6}	$ng \cdot cell^{-1} \cdot h^{-1}$	(37)

82	66	MMP-9 degradation rate	0.0365	h^{-1}	(24)
83	67	Rate of MMP-1 production by pro-inflammatory macrophages	2.0×10^{-8}	$ng·cell^{-1}·h^{-1}$	(38)
84	68	MMP-1 degradation rate	0.3456	h^{-1}	(39)
85	69	Rate of MMP-2 production by pro-inflammatory macrophages	2.0×10^{-7}	$ng·cell^{-1}·h^{-1}$	(38)
86	70	MMP-2 degradation rate	0.3456	h^{-1}	Assumed[†]
87	71	Rate of TIMP-1 production by fibroblasts	1.98×10^{-5}	$ng·cell^{-1}·h^{-1}$	(37)
88	72	TIMP-1 degradation rate	0.6300	h^{-1}	(40)
89	73	Rate of IL-10 production by fibroblasts	1.55×10^{-7}	$ng·cell^{-1}·h^{-1}$	(41)
90	74	Rate of CXCL8 production by fibroblasts	2.5×10^{-7}	$ng·cell^{-1}·h^{-1}$	(41)
91	85	Inverse maximal macrophage density	2.0×10^{-6}	Cells/mL	(24)
92	85	Inverse maximal fibroblast density	2.5×10^{-6}	$Cells/mm^3$	(24)
93	85	Inverse maximal collagen density	4.0×10^{-9}	Ng/mm^3	(24)
94	85	Inverse maximal myofibroblast density	5.0×10^{-6}	$Cells/mm^3$	(24)
95	75	Rate of production of collagen protomer by fibroblasts	833	$ng·cell^{-1}·h^{-1}$	(24, 25)
96	76	Rate of collagen degradation due to MMPs	2.18×10^{-5}	$ng^{-1}·mL·h^{-1}$	(42)
97	77	Rate of collagen degradation due to fibroblast enzymes	6.25×10^{-7}	$ng^{-1}·mL·h^{-1}$	(25)
98	78	Rate of TGF-β production by fibroblasts	1.6×10^{-7}	$ng·cell^{-1}·h^{-1}$	(43)
99	133	Percentage of fibroblast chemotaxis by TGF-β and PDGF	0.1		Assumed[‡]
100	79	Rate of collagen polymerization into intermediate collagen	0.06	h^{-1}	(44)
101	80	Rate of intermediate collagen polymerization into collagen fiber	1.2	h^{-1}	(45)
102	81	Rate of IL-6 production by fibroblasts	2.00×10^{-8}	$ng·cell^{-1}·h^{-1}$	(46)
103	82	Rate of TIMP-1 production by pro-inflammatory macrophages	2.50×10^{-6}	$ng·cell^{-1}·h^{-1}$	(38)
104	83	Rate of MCP-1 production by pro-inflammatory macrophages	4.16×10^{-8}	$ng·cell^{-1}·h^{-1}$	(47)
105	84	MCP-1 degradation rate	2.081	h^{-1}	(48)
106	85	Myofibroblast proliferation rate	1.93×10^{-2}	h^{-1}	(31)
107-108	86	Parameters of the feedback function f_{11} describing the up-regulation of tropocollagen production by MCP-1 $f_{11} = P_{107} MCP\text{-}1^2 + P_{108} MCP\text{-}1$	$P_{107} = -1.6 \times 10^{-7}$ $P_{108} = 0.0123$		(49)
109	87	Rate of VEGF production by pro-inflammatory macrophages	3.09×10^{-9}	$ng·cell^{-1}·h^{-1}$	(50)
110	88	Rate of VEGF production by anti-inflammatory macrophages	3.00×10^{-7}	$ng·cell^{-1}·h^{-1}$	(51)
111	89	Rate of VEGF production by fibroblasts	8.75×10^{-8}	$ng·cell^{-1}·h^{-1}$	(51)
112	90	VEGF degradation rate	0.693	h^{-1}	(52)
113	91	Rate of ANG-1 production by endothelial cells	1.25×10^{-5}	$ng·cell^{-1}·h^{-1}$	(53)
114	92	Rate of ANG-1 production by fibroblasts	6.94×10^{-10}	$ng·cell^{-1}·h^{-1}$	(54)
115	93	ANG-1 degradation rate	0.0042	h^{-1}	(55)
116	94	Rate of ANG-2 production by endothelial cells	2.45×10^{-5}	$ng·cell^{-1}·h^{-1}$	(53)
117	95	Rate of ANG-2 production by fibroblasts	5.5×10^{-10}	$ng·cell^{-1}·h^{-1}$	(54)
118	96	ANG-2 degradation rate	0.1667	h^{-1}	(55)
119	132	Oxygen therapy control	0 or 0.5	% reduction	Introduced by modeler
120	97	Rate of TSP-1 production by pro-inflammatory macrophages	6.00×10^{-5}	$ng·cell^{-1}·h^{-1}$	(56)
121	98	Rate of TSP-1 production by fibroblasts	6.54×10^{-4}	$ng·cell^{-1}·h^{-1}$	(57)
122	99	Rate of TSP-1 production by endothelial cells	5.56×10^{-5}	$ng·cell^{-1}·h^{-1}$	(58)
123	100	TSP-1 degradation rate	1.18	h^{-1}	(59)
124	101	Rate of endostatin production by endothelial cells	4.60×10^{-9}	$ng·cell^{-1}·h^{-1}$	(60)
125	102	Endostatin degradation rate	0.007	h^{-1}	(61)
126	103	Capillary growth rate	0.03	h^{-1}	(62)
127	104	Rate of capillary tip-tip anastomosis	3.70×10^{-8}	$mL·cell^{-1}·h^{-1}$	(63)

128	105	Rate of capillary tip-sprout anastomosis	3.70×10^{-10}	$mL \cdot cell^{-1} \cdot h^{-1}$	(63)
129	103	Maximum capillary density	1.00×10^{-5}	cells/mL	(63)
130	106	Rate of capillary remodeling	0.0542	h^{-1}	(63, 64)
131	107	Rate of oxygen release from blood vessels	2.80	$mmHg \cdot cell^{-1} \cdot h^{-1}$	(64)
132	108	Oxygen degradation rate	1.3	h^{-1}	(64)
133	109	Rate of oxygen consumption by macrophages	6.80×10^{-6}	$mL \cdot cell^{-1} \cdot h^{-1}$	(63)
134	110	Rate of oxygen consumption by fibroblasts	4.50×10^{-6}	$mL \cdot cell^{-1} \cdot h^{-1}$	(63)
135	107	Maximal oxygen concentration	5400	ng/mL	(63)
136	115	Maximal endothelial cell density	1.00×10^{4}	Cells/mL	(61)
137	111	Endothelial cell proliferation rate	0.03	h^{-1}	(65)
138	112	Endothelial cell apoptosis rate	0.09	h^{-1}	(66)
139-140	113	Parameters of the feedback function describing the upregulation of ANG-2 production by VEGF $f_{12} = 1 + \frac{P_{139} VEGF}{1 + VEGF^{P_{140}}}$	$P_{139} = 5.804$ $P_{140} = 1.135$		(67)
141	114	Rate of CXCL8 production by endothelial cells	6.25×10^{-7}	$ng \cdot cell^{-1} \cdot h^{-1}$	(68)
142	115	Rate of endothelial cell chemotaxis	300	mL^{-1}	Assumed§
143	116	Rate of EC tip anastomosis into sprouts	0.0021	h^{-1}	(69)
144	117	Rate of differentiation of blood vessel tip	4.10×10^{-2}	$mL \cdot pg^{-1} \cdot h^{-1}$	(69)
145	118	Rate of IL-6 production by endothelial cells	8.33×10^{-7}	$ng \cdot cell^{-1} \cdot h^{-1}$	(70)
146	119	Rate of VEGF production by endothelial cells	5.20×10^{-10}	$ng \cdot cell^{-1} \cdot h^{-1}$	(61)
147	120	Rate of endothelial cell apoptosis in blood vessels	0.5	fraction	(69)
148	121	Rate of PEDF production by pro-inflammatory macrophages	1.30×10^{-7}	$ng \cdot cell^{-1} \cdot h^{-1}$	(71)
149	122	Rate of PEDF production by endothelial cells	2.50×10^{-6}	$ng \cdot cell^{-1} \cdot h^{-1}$	(72)
150	123	PEDF degradation rate	1.38	h^{-1}	(73)
151	124	Rate of keratinocyte chemotaxis	75	mL^{-1}	Assumed§
152	125	Keratinocyte proliferation rate	486.48	h^{-1}	(74)
153	125	Maximal keratinocyte concentration	1.00×10^{4}	Cells/mL	(75)
154	128	Rate of production of KGF by fibroblasts	3.00×10^{-8}	$ng \cdot cell^{-1} \cdot h^{-1}$	(76)
155	129	KGF degradation rate	0.29	h^{-1}	(75)
156	130	Rate of CXCL1 production by pro-inflammatory macrophages	6.00×10^{-9}	$ng \cdot cell^{-1} \cdot h^{-1}$	(77)
157	131	CXCL1 degradation rate	0.2772	h^{-1}	(78)
158	132	Rate of VEGF production by keratinocytes	1.46×10^{-7}	$ng \cdot cell^{-1} \cdot h^{-1}$	(79)
159	133	Rate of CXCL1 production rate by fibroblasts	2.16×10^{-7}	$ng \cdot cell^{-1} \cdot h^{-1}$	(80)
**Additional parameters used only to model interventions involving TGF-β inhibition and supplementation of FGF-2 and ANG-2**					
160		Rate of binding of TGF-inhibitor with TGF-β	13.35	$nM^{-1} \cdot h^{-1}$	(81)
161		Rate of disassociation of TGF-β and TGF-inhibitor	0.00123	h^{-1}	(81)
162		Concentration of FGF-2 added	0.1 or 0.05	Ng/mL	Introduced by modeler
163		Concentration of ANG-2 added	0.05 or 0.015	Ng/mL	Introduced by modeler

Chemotaxis and protein feedback functions

Effector variable	Affected variable	Chemotaxis function	Parameters	Ref
TGF-β	Active neutrophil concentration	$f_N(TGF\text{-}\beta) = quadratic + linear$ Quadratic: $r_{1q} TGF\text{-}\beta^2 + r_{2q} TGF\text{-}\beta$; $TGF\text{-}\beta \leq 1$ pg/mL Linear: $r_{1l} TGF\text{-}\beta + r_{2l}$; $TGF\text{-}\beta > 1$ pg/mL and ≤ 10 pg/mL	$r_{1q} = -537.30$ $r_{2q} = 698.05$ $r_{1l} = -12.77$ $r_{2l} = 187.78$	(82)

	Pro-inflammatory macrophage concentration	$f_M\,(TGF\text{-}\beta) = quadratic + linear$<br>Quadratic: $r_{3q}TGF\text{-}\beta^2 + r_{4q}TGF\text{-}\beta$; $TGF\text{-}\beta \le 1$ pg/mL<br>Linear: $r_{3l}TGF\text{-}\beta + r_{4l}$; $TGF\text{-}\beta > 1$ pg/mL and ≤ 10 pg/mL	$r_{3q} = -240.29$<br>$r_{4q} = 298.93$<br>$r_{3l} = -0.5926$<br>$r_{4l} = 60.593$	(83)
	Fibroblast concentration (chemotaxis)	$f_F\,(TGF\text{-}\beta) = r_1 e^{(r_2 TGF\text{-}\beta)} + r_3 e^{(r_4 TGF\text{-}\beta)}$	$r_1 = 110.9$<br>$r_2 = -0.01954$<br>$r_3 = -119.5$<br>$r_4 = -0.1376$	(84)
	Fibroblast concentration (proliferation)	$f_{Fpro}\,(TGF\text{-}\beta) = r_5 TGF\text{-}\beta^2 + r_6 TGF\text{-}\beta$	$r_5 = -1.313$<br>$r_6 = 9.42$	
	Myofibroblast concentration	$f_M\,(TGF\text{-}\beta) = r_7 e^{(r_8 TGF\text{-}\beta)} + r_9 e^{(r_{10} TGF\text{-}\beta)}$	$r_7 = 0.003405$<br>$r_8 = 0.005605$<br>$r_9 = -0.00193$<br>$r_{10} = -4.582$	(85)
	Tropocollagen concentration	$f_{COll}\,(TGF\text{-}\beta) = r_{11} TGF\text{-}\beta^3 + r_{12} TGF\text{-}\beta^2 + r_{13} TGF\text{-}\beta + r_{14}$	$r_{11} = 0.0105$<br>$r_{12} = -0.1671$<br>$r_{13} = 0.6417$<br>$r_{14} = 0.2722$	(25)
	Keratinocyte concentration	$f\,(TGF\text{-}\beta) = r_{44} TGF\text{-}\beta + r_{45}$	$r_{44} = 0.0722$<br>$r_{45} = 0.8333$	(74)
PDGF	Active neutrophil concentration	$f_N\,(PDGF) = r_{15} PDGF^2 + r_{16} PDGF$	$r_{15} = -1.02$<br>$r_{16} = 5.9525$	(86)
	Pro-inflammatory macrophage concentration	$f_M\,(PDGF) = r_{17} PDGF^2 + r_{18} PDGF$	$r_{17} = -0.3538$<br>$r_{18} = 11.978$	
	Fibroblast concentration	$f_F\,(PDGF) = r_{19} e^{(r_{20} PDGF)} + r_{21} e^{(r_{22} PDGF)}$	$r_{19} = 5.56$<br>$r_{20} = -0.0013$<br>$r_{21} = -3.167$<br>$r_{22} = -0.5014$	(87)
TNF-α	Pro-inflammatory macrophage concentration	$f_M\,(TNF\text{-}\alpha) = r_{23} TNF\text{-}\alpha^2 + r_{24} TNF\text{-}\alpha$	$r_{23} = -0.3164$<br>$r_{24} = 10.708$	(88)
	Fibroblast concentration	$f_F\,(TNF\text{-}\alpha) = r_{25} e^{(r_{26} TNF\text{-}\alpha)} + r_{27} e^{(r_{28} TNF\text{-}\alpha)}$	$r_{25} = 2.52$<br>$r_{26} = -0.0006$<br>$r_{27} = -2.453$<br>$r_{28} = -0.00446$	(89)
	ANG-2	$f_{ANG\text{-}2}\,(TNF\text{-}\alpha) = r_{46} e^{(r_{47} TNF\text{-}\alpha)} + r_{48} e^{(r_{49} TNF\text{-}\alpha)}$	$r_{46} = 0.000222$<br>$r_{47} = -9.743$<br>$r_{48} = 0.1529$<br>$r_{49} = -0.1252$	(90)
IL-6	ANG-2	$f_{ANG\text{-}2}\,(IL\text{-}6) = r_{50} e^{(r_{51} IL\text{-}6)} + r_{52} e^{(r_{53} IL\text{-}6)}$	$r_{50} = 0.000817$<br>$r_{51} = -8.163$<br>$r_{52} = 0.1719$<br>$r_{53} = -0.2825$	(90)
IL-1β	Keratinocyte	$f_K\,(IL\text{-}1\beta) = r_{54} IL\text{-}1\beta + r_{55}$	$r_{54} = 0.0678$<br>$r_{55} = 0.8556$	(74)
*CXCL*1	Active neutrophil concentration	$f_N\,(CXCL1) = r_{56} CXCL1^2 + r_{57} CXCL1$	$r_{56} = -4.2303$<br>$r_{57} = 632.37$	(91)
*CXCL*8	Active neutrophil concentration	$f_N\,(CXCL8) = r_{29} CXCL8^2 + r_{30} CXCL8$	$r_{29} = -0.1045$<br>$r_{30} = 13.678$	(92)
MIP-1*α*	Pro-inflammatory macrophage concentration	$f_{Mpro}\,(MIP\text{-}1\alpha) = r_{31} MIP\text{-}1\alpha^3 + r_{32} MIP\text{-}1\alpha^2 + r_{33} MIP\text{-}1\alpha$	$r_{31} = 0.0006$<br>$r_{32} = -0.1481$<br>$r_{33} = 11.36$	

FGF-2	Fibroblast concentration	$f_F(FGF\text{-}2) = r_{34}FGF\text{-}2^2 + r_{35}FGF\text{-}2$	r_{34} = –0.0077<br>r_{35} = 0.8591	(93)
FGF-2	Endothelial cell concentration	$f_{EC}(FGF\text{-}2) = r_{58}FGF\text{-}2^2 + r_{59}FGF\text{-}2$	r_{58} = –0.002<br>r_{59} = 0.8119	(93)
Fibronectin	Fibroblast concentration	$f_F(fibnec) = r_{36}e^{(r_{37}\,fibnec)} + r_{38}e^{(r_{39}\,fibnec)}$	r_{36} = 15.05<br>r_{37} = -1.3×10^{-5}<br>r_{38} = –15.65<br>r_{39} = –0.00183	(94)
MCP-1	Pro-inflammatory macrophage concentration	$f(MCP\text{-}1) = quadratic + linear$<br>Quadratic: $r_{5q}MCP\text{-}1^2 + r_{6q}MCP\text{-}1$; *MCP*-1 ≤12 pg/mL<br>Linear: $r_{5l}MCP\text{-}1 + r_{6l}$; *MCP*-1 >12 pg/mL	r_{5q} = –8.152<br>r_{6q} = 110<br>r_{5l} = –0.0586<br>r_{6l} = 126.1	(95)
$Coll_{fib}$	Tropocollagen	$f(coll_{fib}) = r_{40}coll_{fib}^3 + r_{41}coll_{fib}^2 + r_{42}coll_{fib} + r_{43}$	r_{40} = -4.3×10^{-10}<br>r_{41} = 9×10^{-7}<br>r_{42} = 0.00055<br>r_{43} = 0.13	(24)
VEGF	Endothelial cell concentration	$f_{EC}(VEGF) = r_{60}VEGF^2 + r_{61}VEGF$	r_{60} = –0.6704<br>r_{61} = 32.811	(96)
VEGF	Endothelial cell proliferation	$f_{EC\_prolif}(VEGF) = r_{62}VEGF^2 + r_{63}VEGF + 1$	r_{62} = -3.0×10^{-5}<br>r_{63} = 0.0147	(97)
VEGF	Endothelial cell apoptosis	$f_{EC\_apop}(VEGF) = r_{64}VEGF^2 + r_{65}VEGF + 1$	r_{64} = 0.002<br>r_{65} = 0.0633	(98)
VEGF	Keratinocytes	$f_K(VEGF) = r_{66}VEGF^2 + r_{67}VEGF$	r_{66} = –0.1321<br>r_{67} = 18.271	(99)
ANG-1	CXCL8	$f_{CXCL8}(ANG\text{-}1) = r_{68}ANG\text{-}1 + 1$	r_{68} = 0.0062	(100)
ANG-2	Endothelial cell concentration	$f_{EC}(ANG\text{-}2) = r_{69}ANG\text{-}2^2 + r_{70}ANG\text{-}2$	r_{69} = –0.06<br>r_{70} = 7.03	(101)
PEDF	Endothelial cell concentration (proliferation)	$f_{EC\_prolif}(PEDF) = r_{71}PEDF + r_{72}$	r_{71} = -3.0×10^{-5}<br>r_{72} = 1.0	(102)
PEDF	Endothelial cell concentration (apoptosis)	$f_{EC}(PEDF) = r_{73}PEDF^2 + r_{74}PEDF + r_{75}$	r_{73} = -4.0×10^{-7}<br>r_{74} = 0.0018<br>r_{75} = 1.0	(102)
PEDF	VEGF (keratinocytes)	$f_{VEGF}(PEDF) = r_{76}PEDF + r_{77}$	r_{76} = –0.0173<br>r_{77} = 1.0	(103)
Oxygen	Tropocollagen (fibroblasts)	$f_{Coll\_F}(O) = r_{78}O + r_{79}$	r_{78} = -6.0×10^{-5}<br>r_{79} = 1.5539	(104)
Oxygen	Tropocollagen (myofibroblasts)	$f_{Coll\_MyoF}(O) = r_{80}O + r_{81}$	r_{80} = 1.0×10^{-4}<br>r_{81} = 0.0688	(105)
Oxygen	VEGF (pro-inflammatory macrophages)	$f_{VEGF}(O) = r_{82}O + r_{83}$	r_{82} = –0.0014<br>r_{83} = 1.3528	(50)
Oxygen	VEGF (fibroblasts)	$f_{VEGF\_F}(O) = r_{84}O^2 + r_{85}O + r_{86}$	r_{84} = 8.0×10^{-7}<br>r_{85} = –0.0035<br>r_{86} = 4.4965	(106)
Oxygen	PEDF	$f_{PEDF}(O) = r_{87}O^2 + r_{88}O + r_{89}$	r_{87} = -2.0×10^{-8}<br>r_{88} = 0.0003<br>r_{89} = 0.1444	(107)

Endostatin	Endothelial cell concentration	$f_{EC}(endo) = r_{90}endo^2 + r_{91}endo + r_{92}$	$r_{90} = 2.0 \times 10^{-6}$<br>$r_{91} = 0.0023$<br>$r_{92} = 1.0073$	(108)
TSP-1	Endothelial cell concentration	$f_{EC}(TSP\text{-}1) = r_{93}TSP\text{-}1 + r_{94}$	$r_{93} = -0.0003$<br>$r_{94} = 0.8692$	(109)
**Mechanical stress effect functions**				
Mechanical strain	CXCL8	$f_{CXCL8}(M_{strain}) = r_{95}M_{strain} + 1$	$r_{95} = 0.0481$	(110)
	IL-6	$f_{IL\text{-}6}(M_{strain}) = r_{96}M_{strain} + 1$	$r_{96} = 0.0429$	(46)
	TGF-β	$f_{TGF\text{-}\beta}(M_{strain}) = r_{97}M_{strain} + r_{98}$	$r_{97} = 0.08$<br>$r_{98} = 0.9767$	(111)
	Tropocollagen	$f_C(M_{strain}) = r_{99}M_{strain} + r_{100}$	$r_{99} = 0.0313$<br>$r_{100} = 0.995$	
	Fibroblast	$f_F(M_{strain}) = r_{101}M_{strain} + r_{102}$	$r_{101} = 0.0099$<br>$r_{102} = 0.9928$	

Model variables and equations

Model variables: volume concentrations for different cell types and molecular species

N_{act}	Active neutrophils	$TGF\text{-}\beta$	Transforming growth factor-β
N_{apop}	Apoptotic neutrophils	$PDGF$	Platelet-derived growth factor
M_{pro}	Pro-inflammatory macrophages	$IL\text{-}1\beta$	Interleukin-1β
M_{anti}	Anti-inflammatory macrophages	$IL\text{-}6$	Interleukin-6
P	Platelets	$MIP\text{-}1\alpha$	Macrophage inflammatory protein-1α
$CXCL8$	Chemokine $CXCL8$	$MIP\text{-}2$	Macrophage inflammatory protein-2
$IL\text{-}12$	Interleukin-12	$IP\text{-}10$	Interferon-γ-induced protein 10
$IL\text{-}10$	Interleukin-10	$TNF\text{-}\alpha$	Tumor necrosis factor-α
F	Fibroblasts	$MyoF$	Myofibroblasts
$Coll$	Tropocollagen	$Coll_{fib}$	Collagen fiber
$Coll_{int}$	Collagen fibril	$Fibnec$	Fibronectin
FGF	Fibroblast growth factor	$MMP\text{-}9$	Matrix metalloproteinase-9
$MMP\text{-}1$	Matrix metalloproteinase-1	$MMP\text{-}2$	Matrix metalloproteinase-2
$MCP\text{-}1$	Monocyte chemo attractant protein-1	$TIMP\text{-}1$	Tissue inhibitor of matrix metalloproteinase-1
$VEGF$	Vascular endothelial growth factor	$ANG\text{-}1$	Angiopoietin-1
$ANG\text{-}2$	Angiopoietin-2	$endo$	Endostatin
O	Oxygen	$TSP\text{-}1$	Thrombospondin-1
EC	Endothelial cells	$capsprout$	Capillary sprouts
$PEDF$	Pigment epithelium-derived factor	K	Keratinocytes
KGF	Keratinocyte growth factor	$CXCL1$	Chemokine CXCL1
**Additional variables added to the model and used only to simulate interventions involving TGF-β inhibition**			
$ITGF\text{-}\beta$	TGF-β inhibitor	$I\_TGF\text{-}\beta$	Inhibitor bound TGF- β

Model equations[†]

$$dP/dt = -k_1 P;$$

$$dN_{act}/dt = k_3\{f_N(TGF\text{-}\beta) + f_N(PDGF) + f_N(CXCL8)\} - k_4 N_{act};$$

$$dN_{apopt}/dt = k_4 N_{act} - \frac{k_5 N_{apop}}{k_6 + N_{apop}} M_{pro};$$

$$dM_{pro}/dt = k_7\{f_M(TGF\text{-}\beta(t-12)) + f_M(PDGF) + f_M(TNF\text{-}\alpha) + f_M(MIP\text{-}1\alpha) + f_M(MCP\text{-}1)\}$$

$$-\left(\frac{k_8 N_{apop}}{k_9 + N_{apop}}\right) M_{pro} - k_{10}\left(1 - 4\frac{k_8 N_{apop}}{k_9 + N_{apop}}\right) M_{pro};^*$$

$$dM_{anti}/dt = ((k_8 N_{apop})/(k_9 + N_{apop}))M_{pro} - k_{10} M_{anti};$$

$$dTNF\text{-}\alpha/dt = k_{44} N_{act} + k_{17} f_1 f_4 f_6 M_{pro} + k_{18} M_{anti} - k_{19} TNF\text{-}\alpha;$$

$$dTGF\text{-}\beta/dt = k_2 P + k_{11} M_{pro} + k_{12} M_{anti} + k_{98} f_{TGF\beta}(M_{strain})(F + 2MyoF) - k_{13} TGF\text{-}\beta;$$

$$dPDGF/dt = k_{14} M_{pro} + k_{15} M_{anti} - k_{16} PDGF;$$

$$dIL\text{-}1\beta/dt = k_{45} N_{act} + k_{20} f_3 f_5 f_7 M_{pro} + k_{21} M_{anti} - k_{22} IL\text{-}1\beta;$$

$$dCXCL8/dt = k_{29} M_{pro} + k_{30} M_{anti} + k_{90} f_{CXCL8}(M_{strain})F + k_{141} f_{CXCL8}(ANG\text{-}1)EC - k_{31} CXCL8;$$

$$dIL\text{-}6/dt = k_{46} N_{act} + k_{23} f_2(1 + f_9)M_{pro} + k_{24} M_{anti} + k_{102} f_{IL6}(M_{strain})F + k_{145} EC - k_{27} IL\text{-}6;$$

$$dIL\text{-}10/dt = k_{26}(1 + f_{10})M_{pro} + k_{27} M_{anti} + k_{89} F - k_{28} IL\text{-}10;$$

$$dIL\text{-}12/dt = k_{32} f_8 M_{pro} - k_{33} IL\text{-}12;$$

$$dMIP\text{-}1\alpha/dt = k_{34} M_{pro} + k_{35} M_{anti} - k_{36} MIP\text{-}1\alpha;$$

$$dMIP\text{-}2/dt = k_{37} M_{pro} + k_{38} M_{anti} - k_{39} MIP\text{-}2;$$

$$dIP\text{-}10/dt = k_{40} M_{pro} + k_{41} M_{anti} - k_{42} IP\text{-}10;$$

$$dF/dt = k_{56}[k_{99}\{f_F(TGF\text{-}\beta) + f_F(PDGF)\} + f_F(Fibnec) + f_F(FGF\text{-}2) + f_F(TNF\text{-}\alpha)]$$

$$+k_{57} f_F(M_{strain}) f_{F_{pro}}(TGF\text{-}\beta)\{(1 - k_{91}(M_{pro} + M_{anti}) - k_{92} F - k_{94} MyoF - k_{93} Coll_{fib})\}F - f_{myoF}(TGF\text{-}\beta)F - k_{58} F;$$

$$dMyoF/dt = f_{MyoF}(TGF\text{-}\beta)F + k_{106}(1 - k_{91}(M_{pro} + M_{anti}) - k_{92} F - k_{94} MyoF - k_{93} Coll_{fib})MyoF - k_{74} MyoF;$$

$$dColl/dt = k_{95}(1 + f_{11}) f_{Coll}(TGF\text{-}\beta) f_C(M_{strain}) f_{Coll}(Coll_{fib})(f_{Coll\_F}(O)F + 2 f_{Coll\_MyoF}(O)MyoF) - k_{100} Coll + k_{96}(MMP\text{-}1 + MMP\text{-}2)Coll_{fib};$$

$$dColl_{int}/dt = k_{100} Coll_{pro} - k_{101} Coll_{int};$$

$$dColl_{fib}/dt = k_{101} Coll_{int} - k_{96}(MMP\text{-}1 + MMP\text{-}2)Coll_{fib} - k_{97}(F + 2MyoF)Coll_{fib};$$

$$dFibnec/dt = k_{75} M_{pro} + k_{76} F - k_{77} Fibnec;$$

$$dFGF\text{-}2/dt = k_{78} M_{pro} + k_{43} M_{anti} - k_{79} FGF\text{-}2;$$

$$dMMP\text{-}1/dt = k_{83} M_{pro} - k_{84} MMP\text{-}1;$$

$$dMMP\text{-}2/dt = k_{85} M_{pro} - k_{86} MMP\text{-}2;$$

$$dMMP\text{-}9/dt = k_{80} M_{pro} + k_{81} F - k_{82} MMP\text{-}9;$$

$$dTIMP\text{-}1/dt = k_{103} M_{pro} + k_{87} F - k_{88} TIMP\text{-}1;$$

$$dMCP\text{-}1/dt = k_{104} M_{pro} - k_{105} MCP\text{-}1;$$

$$dVEGF/dt = k_{109} f_{VEGF}(O)M_{pro} + k_{110} M_{anti} + k_{111} f_{VEGF\_F}(O)F + k_{146} EC - k_{112} VEGF;$$

$$dANG\text{-}1/dt = k_{113} F + k_{114} EC - k_{115} ANG\text{-}1;$$

$$dANG\text{-}2/dt = k_{117} F + k_{116} f_{ANG\text{-}2}(IL\text{-}6) f_{ANG\text{-}2}(TNF\text{-}\alpha)EC - k_{118} ANG\text{-}2;$$

$$dTSP\text{-}1/dt = k_{120} M_{pro} + k_{121} F + k_{122} EC - k_{123} TSP\text{-}1;$$

$$dendo / dt = k_{124} EC - k_{125} endo;$$

$$dEC / dt = H(k_{136} - EC) k_{142} f_{EC}(TSP\text{-}1)[f_{EC}(FGF\text{-}2) + f_{EC}(VEGF) + f_{EC}(ANG\text{-}2)] + k_{137} f_{EC}(endo) f_{EC\_prolif}(VEGF) EC - k_{138} f_{EC}(VEGF) EC;$$

$$dcapsprout / dt = H(k_{129} - capsprout)[(k_{127} EC + k_{128} capsprout)] EC + [(k_{144} + k_{143} VEGF)] EC - k_{147} f_{capsprout}(VEGF) k_{138} capsprout$$

$$+ k_{130} capsprout[1 - (capsprout / k_{129})];$$

$$dO / dt = (1 + k_{119}) k_{131} B - [k_{134} F + k_{133}(M_{pro} + M_{anti})] O - k_{132} O;$$

$$dPEDF / dt = k_{148} f_{PEDF}(O) F + k_{149} f_{PEDF}(VEGF) K - k_{150} PEDF;$$

$$dK / dt = k_{151} f_K(VEGF) + k_{152} f_K(TGF\text{-}\beta) f_K(IL\text{-}1\beta)(1 - K / k_{133});$$

$$dKGF / dt = k_{154} F - k_{155} KGF;$$

$$dCXCL1 / dt = k_{156} M_{pro} + k_{159} F - k_{157} CXCL1;$$

$$dITGF\text{-}\beta / dt = -k_{160} ITGF\text{-}\beta TGF\text{-}\beta + k_{161} ITGF\text{-}\beta;$$

$$dI\_TGF\text{-}\beta / dt = k_{160} ITGF\text{-}\beta TGF\text{-}\beta - k_{161} I\_TGF\text{-}\beta;$$

§This parameter was used as a multiplier for the chemotaxis functions for neutrophils, macrophages, fibroblasts, endothelial cells, and keratinocytes to compensate for the difference between the units reported in the experimental data (cells/field) and the concentration units used in our model (cells/mL). These parameters (i.e., k_3, k_7, k_{56}, $k_{142,\text{ and}}$ k_{151}) reflect the differences between the volumes of cell medium used in experiments (with values in the microliter range) and the volume units (mL) used in our model simulations.

*Values for these rates, which characterize the production of pro-inflammatory mediators by anti-inflammatory macrophages in our model, were not available in the literature. To parameterize our model, we assumed that these rates are equal to 10% of the rates of production of the corresponding mediators by pro-inflammatory macrophages.

†Our value for the MMP-2 degradation rate was assumed to be the same as the MMP-1 degradation rate, because they belong to the same family of collagenases.

‡Because TGF-β and PDGF are the primary chemoattractants for macrophages and neutrophils, their contribution to fibroblast chemotaxis was assumed to be 10% of their calculated chemotaxis rates in vitro [i.e., the functions $f_F(TGF\text{-}\beta)$ and $f_F(PDGF)$].

NOTE: parameters 47-55, 59-69, 107-108, and 139-140 are dimensionless parameters that describe positive and negative regulatory feedback functions for the production rates of certain molecular mediators and tropocollagen. The form of the feedback functions was chosen by fitting different types of functions (e.g., linear, exponential, and polynomial) to experimental data and selecting the function that provided the best fit.

NOTE: The r_i parameters in the chemotaxis functions and the functions representing feedback of molecular mediators on the increase or inhibition of other molecular mediators were not included in the main parameter list (P#1 to P#163), because they do not explicitly represent rates of biological processes.

†In the model equations, the rate constants for different modeled processes are designated by the letter "*k*" The subscripts following the "*k*" correspond to the parameter number P# (see left column) used to represent that particular rate constant in our implementation of the model. The quantities f_i in the model equations designate functions for cytokine feedback, chemotaxis, and mechanical stress and are defined in the table above.

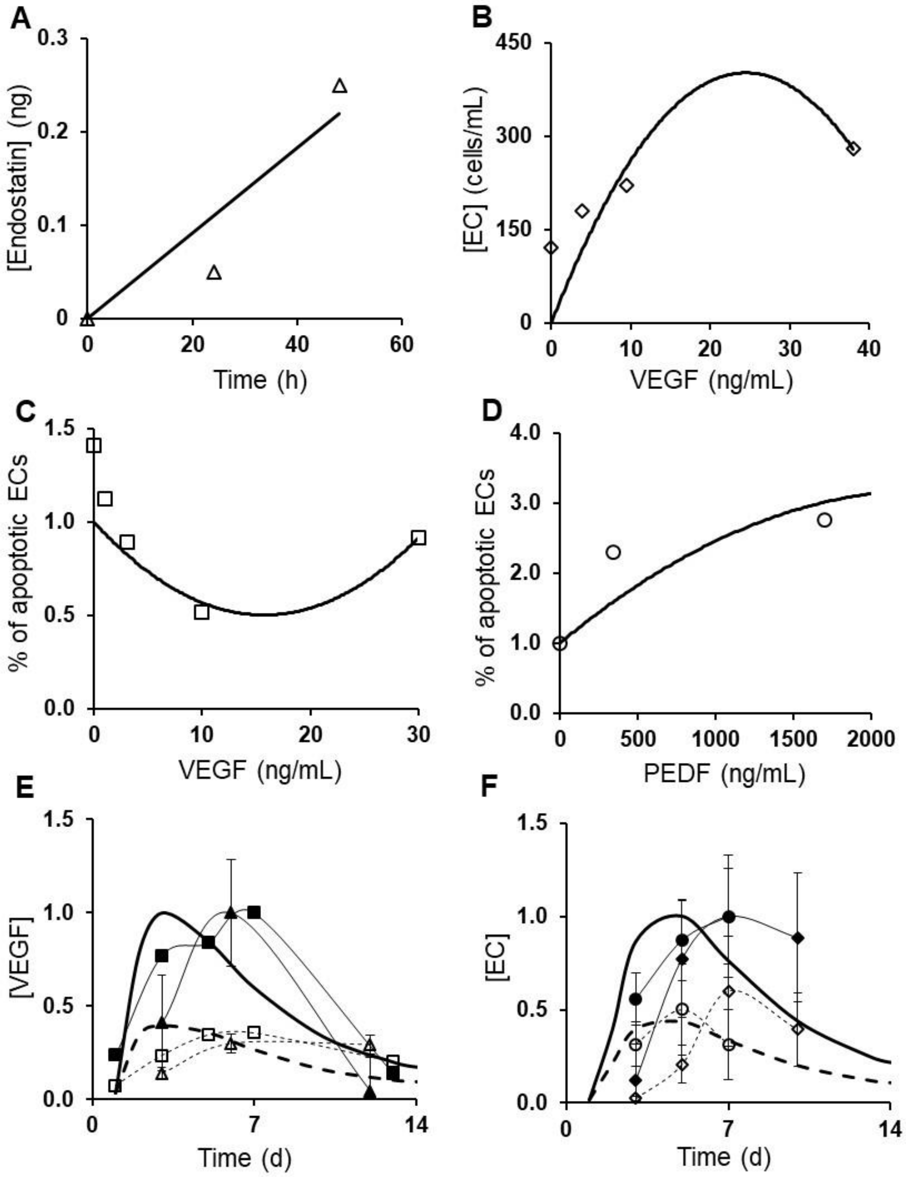

Figure S1. Parameter-value estimation by experimental-data fitting. A: Experimental data (open triangles) on the production of endostatin by endothelial cells were obtained from Ref. (60) and approximated using linear regression analysis (solid line). The slope of the regression line represents the estimated endostatin production rate. B: Experimental data (open diamonds) on endothelial cell chemotaxis induced by VEGF were obtained from Ref. (96). The migration-rate functions were obtained by fitting the experimental data using a quadratic function (solid lines). Brackets designate concentration. Day zero indicates the day of injury initiation. C and D: Experimental data on the percentage change in apoptotic endothelial cell concentration induced

by varying concentrations of VEGF (open squares) and PEDF (open circles) were obtained from Refs. (102) and (98), respectively. Solid lines show the fitting of the experimental data with quadratic functions These functions are listed as $f_{EC\_apop}(VEGF)$ and $f_{EC}(PEDF)$ in Table S2. E and F: Solid and dashed lines show model simulations for normal and impaired angiogenesis, respectively; solid symbols show experimental data from wounds of wild-type mice; open symbols show experimental data from wounds of diabetic mice. Brackets designate normalized concentration. Day 0 indicates the day of injury initiation. Experimental data were obtained from previously published experimental studies in mouse and dog wounds: solid and open circles (112), solid and open diamonds (113), solid and open triangles (114), solid and open squares (115). These experimental data sets of impaired angiogenesis were different from the ones used for validation of our model simulations of impaired angiogenesis (Fig. 3 in the main text). We varied the default values of two model parameters, namely, the production rate of VEGF by anti-inflammatory macrophages and EC apoptosis rate, until the simulated time courses of VEGF and ECs were close to their corresponding time course data obtained from diabetic mouse wounds (open symbols). For meaningful comparisons between the model predictions and experimental data, normalization was necessary because of the differences in reporting units between the experimental data and model simulations. For each model-predicted time course, normalization was performed by dividing that time course by its maximal value. For each time course obtained from available experimental data, we first extracted the means and standard deviations (based on the sample size information provided in each study) at each time point. Then, we divided the mean and standard deviation values by the maximum mean value in the time course.

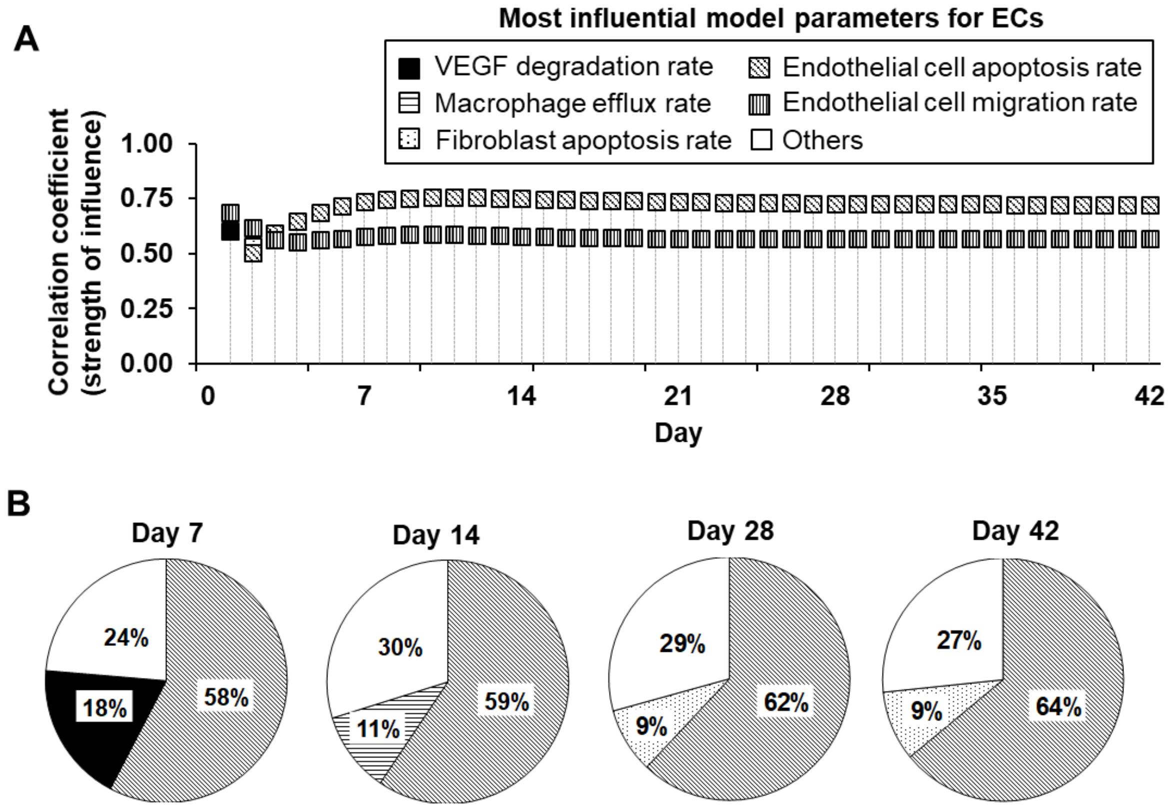

Figure S2. The most influential model parameters for ECs predicted by the model: temporal dependencies of the influence strength. Patterns represent a given model parameter. Vertical stripes: EC migration rate; solid black: VEGF degradation rate; diagonal stripes: EC apoptosis rate; horizontal stripes: macrophage efflux rate; and dotted: fibroblast apoptosis rate. A: Patterned squares show the PRCCs (reflecting the strength of influence) between the most influential model parameters and EC concentration on different days in the model-predicted time course. The model parameters for which the PRCCs were above 0.5 with $P \leq 0.05$ were identified as the most influential model parameters for a given model variable. B: Pie chart shows the percentage of 51,175 simulated wound-healing scenarios for which the ECs exhibited the *highest sensitivity* to a given parameter among the 159 model parameters. Open square (labeled as others) represents the parameters (among the 159 model parameters) to which ECs exhibited the *highest* sensitivity in a fraction of simulations that was too small (< 10%) to be considered as influential.

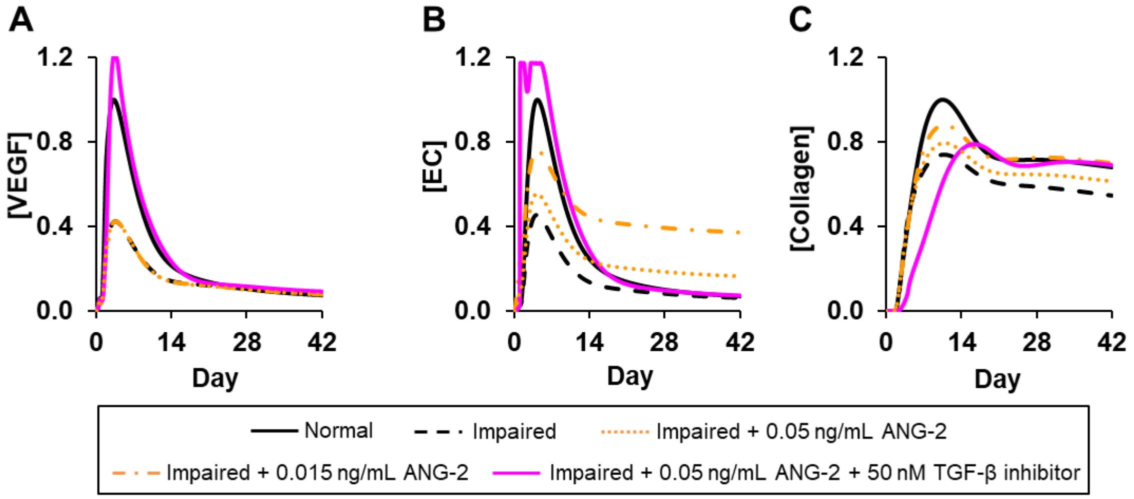

Figure S3. Angiogenesis regulation by modulation of model-identified proteins targets. Shown are the model-predicted (A) VEGF, (B) EC, and (C) collagen concentration time courses during normal angiogenesis (solid black lines), impaired angiogenesis (dashed black lines), and three simulated intervention scenarios, namely, supplementing ANG-2 at concentrations of 0.05 ng/mL (dotted orange lines) and 0.015 ng/mL (dash-dot orange lines), and the simultaneous addition of TGF-β inhibitor at a concentration of 50 nM and supplementation of ANG-2 at a concentration of 0.05 ng/mL (solid pink lines). We simulated impaired angiogenesis by decreasing the VEGF production rate via anti-inflammatory macrophages by 3-fold and increasing the endothelial cell apoptosis rate by 1.2-fold. All interventions were introduced 1 hour post-wounding.

REFERENCES

1. Mosnier, L. O., P. A. von dem Borne, J. C. Meijers, and B. N. Bouma. 1998. Plasma TAFI levels influence the clot lysis time in healthy individuals in the presence of an intact intrinsic pathway of coagulation. *Thromb Haemost* 80: 829-835.
2. Grainger, D. J., L. Wakefield, H. W. Bethell, R. W. Farndale, and J. C. Metcalfe. 1995. Release and activation of platelet latent TGF-beta in blood clots during dissolution with plasmin. *Nat Med* 1: 932-937.
3. Wakefield, L. M., D. M. Smith, K. C. Flanders, and M. B. Sporn. 1988. Latent transforming growth factor-beta from human platelets. A high molecular weight complex containing precursor sequences. *J Biol Chem* 263: 7646-7654.
4. Reynolds, A., J. Rubin, G. Clermont, J. Day, Y. Vodovotz, and G. Bard Ermentrout. 2006. A reduced mathematical model of the acute inflammatory response: I. Derivation of model and analysis of anti-inflammation. *J Theor Biol* 242: 220-236.
5. Newman, S. L., J. E. Henson, and P. M. Henson. 1982. Phagocytosis of senescent neutrophils by human monocyte-derived macrophages and rabbit inflammatory macrophages. *J Exp Med* 156: 430-442.
6. Cobbold, C. A., and J. A. Sherratt. 2000. Mathematical modelling of nitric oxide activity in wound healing can explain keloid and hypertrophic scarring. *J Theor Biol* 204: 257-288.
7. Cassatella, M. A. 1995. The production of cytokines by polymorphonuclear neutrophils. *Immunol Today* 16: 21-26.
8. Fadok, V. A., D. L. Bratton, A. Konowal, P. W. Freed, J. Y. Westcott, and P. M. Henson. 1998. Macrophages that have ingested apoptotic cells in vitro inhibit proinflammatory cytokine production through autocrine/paracrine mechanisms involving TGF-beta, PGE2, and PAF. *J Clin Invest* 101: 890-898.
9. Werner, S., and R. Grose. 2003. Regulation of wound healing by growth factors and cytokines. *Physiol Rev* 83: 835-870.
10. Chu, X., J. Newman, B. Park, S. Nares, G. Ordonez, and A. M. Iacopino. 1999. In vitro alteration of macrophage phenotype and function by serum lipids. *Cell Tissue Res* 296: 331-337.

11. Bhandari, B., G. Grandaliano, and H. E. Abboud. 1994. Platelet-derived growth factor (PDGF) BB homodimer regulates PDGF A- and PDGF B-chain gene transcription in human mesangial cells. *Biochem J* 297 (Pt 2): 385-388.
12. Byrne, A., and D. J. Reen. 2002. Lipopolysaccharide induces rapid production of IL-10 by monocytes in the presence of apoptotic neutrophils. *J Immunol* 168: 1968-1977.
13. Wong, S., R. C. Schwartz, and J. J. Pestka. 2001. Superinduction of TNF-alpha and IL-6 in macrophages by vomitoxin (deoxynivalenol) modulated by mRNA stabilization. *Toxicology* 161: 139-149.
14. Klapproth, J., J. Castell, T. Geiger, T. Andus, and P. C. Heinrich. 1989. Fate and biological action of human recombinant interleukin 1 beta in the rat in vivo. *Eur J Immunol* 19: 1485-1490.
15. Smythies, L. E., M. Sellers, R. H. Clements, M. Mosteller-Barnum, G. Meng, W. H. Benjamin, J. M. Orenstein, and P. D. Smith. 2005. Human intestinal macrophages display profound inflammatory anergy despite avid phagocytic and bacteriocidal activity. *J Clin Invest* 115: 66-75.
16. Verreck, F. A., T. de Boer, D. M. Langenberg, L. van der Zanden, and T. H. Ottenhoff. 2006. Phenotypic and functional profiling of human proinflammatory type-1 and anti-inflammatory type-2 macrophages in response to microbial antigens and IFN-gamma- and CD40L-mediated costimulation. *J Leukoc Biol* 79: 285-293.
17. Shi, M. M., J. J. Godleski, and J. D. Paulauskis. 1995. Molecular cloning and posttranscriptional regulation of macrophage inflammatory protein-1 alpha in alveolar macrophages. *Biochem Biophys Res Commun* 211: 289-295.
18. Bajetta, E., M. Del Vecchio, R. Mortarini, R. Nadeau, A. Rakhit, L. Rimassa, C. Fowst, A. Borri, A. Anichini, and G. Parmiani. 1998. Pilot study of subcutaneous recombinant human interleukin 12 in metastatic melanoma. *Clin Cancer Res* 4: 75-85.
19. Lucas, M., L. M. Stuart, J. Savill, and A. Lacy-Hulbert. 2003. Apoptotic cells and innate immune stimuli combine to regulate macrophage cytokine secretion. *J Immunol* 171: 2610-2615.
20. Chong, I. W., S. R. Lin, J. J. Hwang, M. S. Huang, T. H. Wang, M. S. Tsai, J. J. Hou, and J. D. Paulauskis. 2000. Expression and regulation of macrophage inflammatory protein-2 gene by vanadium in mouse macrophages. *Inflammation* 24: 127-139.

21. Vockerodt, M., D. Pinkert, S. Smola-Hess, A. Michels, R. M. Ransohoff, H. Tesch, and D. Kube. 2005. The Epstein-Barr virus oncoprotein latent membrane protein 1 induces expression of the chemokine IP-10: Importance of mRNA half-life regulation. *Int. J. Cancer* 114: 598-605.
22. Chernykh, E., E. Y. Shevela, L. Sakhno, M. Tikhonova, Y. Petrovsky, and A. Ostanin. 2010. The generation and properties of human M2-like macrophages: potential candidates for CNS repair. *Cell Ther Transplant* 2: 1-8.
23. Thomassen, M. J., L. T. Divis, and C. J. Fisher. 1996. Regulation of human alveolar macrophage inflammatory cytokine production by interleukin-10. *Clin Immunol Immunopathol* 80: 321-324.
24. Jin, Y. F., H. C. Han, J. Berger, Q. Dai, and M. L. Lindsey. 2011. Combining experimental and mathematical modeling to reveal mechanisms of macrophage-dependent left ventricular remodeling. *BMC Syst Biol* 5: 60.
25. Waugh, H. V., and J. A. Sherratt. 2007. Modeling the effects of treating diabetic wounds with engineered skin substitutes. *Wound Repair Regen* 15: 556-565.
26. Chantry, D., M. Turner, E. Abney, and M. Feldmann. 1989. Modulation of cytokine production by transforming growth factor-beta. *J Immunol* 142: 4295-4300.
27. Schindler, R., J. Mancilla, S. Endres, R. Ghorbani, S. C. Clark, and C. A. Dinarello. 1990. Correlations and interactions in the production of interleukin-6 (IL-6), IL-1, and tumor necrosis factor (TNF) in human blood mononuclear cells: IL-6 suppresses IL-1 and TNF. *Blood* 75: 40-47.
28. Ma, X., J. Sun, E. Papasavvas, H. Riemann, S. Robertson, J. Marshall, R. T. Bailer, A. Moore, R. P. Donnelly, G. Trinchieri, and L. J. Montaner. 2000. Inhibition of IL-12 production in human monocyte-derived macrophages by TNF. *J Immunol* 164: 1722-1729.
29. Turner, M., D. Chantry, and M. Feldmann. 1990. Transforming growth factor beta induces the production of interleukin 6 by human peripheral blood mononuclear cells. *Cytokine* 2: 211-216.
30. Maeda, H., H. Kuwahara, Y. Ichimura, M. Ohtsuki, S. Kurakata, and A. Shiraishi. 1995. TGF-beta enhances macrophage ability to produce IL-10 in normal and tumor-bearing mice. *J Immunol* 155: 4926-4932.

31. Olsen, L., J. A. Sherratt, and P. K. Maini. 1995. A mechanochemical model for adult dermal wound contraction and the permanence of the contracted tissue displacement profile. *J Theor Biol* 177: 113-128.
32. Rennard, S. I., G. W. Hunninghake, P. B. Bitterman, and R. G. Crystal. 1981. Production of fibronectin by the human alveolar macrophage: mechanism for the recruitment of fibroblasts to sites of tissue injury in interstitial lung diseases. *Proc Natl Acad Sci U S A* 78: 7147-7151.
33. Varani, J., and S. Chakrabarty. 1990. Modulation of fibronectin synthesis and fibronectin binding during transformation and differentiation of mouse AKR fibroblasts. *J Cell Physiol* 143: 445-454.
34. Reilly, J. T., B. A. McVerry, and M. J. Mackie. 1983. Fibronectin in blood products--an in vitro and in vivo study. *J Clin Pathol* 36: 1377-1381.
35. Beenken, A., and M. Mohammadi. 2009. The FGF family: biology, pathophysiology and therapy. *Nat Rev Drug Discov* 8: 235-253.
36. James, B. P., W. Sicheng, P.-H. Qiang, and Q.-J. Marianne. 2005. Secretion of matrix metalloproteinase-9 by macrophages, in vitro, in response to Helicobacter pylori. *FEMS Immunol Med Microbiol* 45: 159-169.
37. Pardo, A., M. Selman, R. Ramirez, C. Ramos, M. Montano, G. Stricklin, and G. Raghu. 1992. Production of collagenase and tissue inhibitor of metalloproteinases by fibroblasts derived from normal and fibrotic human lungs. *Chest* 102: 1085-1089.
38. Serra, R., A. G. Al-Saidi, N. Angelov, and S. Nares. 2010. Suppression of LPS-induced matrix-metalloproteinase responses in macrophages exposed to phenytoin and its metabolite, 5-(p-hydroxyphenyl-), 5-phenylhydantoin. *J Inflamm* 7: 48.
39. Bedair, H., T. T. Liu, J. L. Kaar, S. Badlani, A. J. Russell, Y. Li, and J. Huard. 2007. Matrix metalloproteinase-1 therapy improves muscle healing. *J Appl Physiol (1985)* 102: 2338-2345.
40. Batra, J., J. Robinson, C. Mehner, A. Hockla, E. Miller, D. C. Radisky, and E. S. Radisky. 2012. PEGylation extends circulation half-life while preserving in vitro and in vivo activity of tissue inhibitor of metalloproteinases-1 (TIMP-1). *PLOS ONE* 7: e50028.

41. Morandini, A. C., C. R. Sipert, E. S. Ramos-Junior, D. T. Brozoski, and C. F. Santos. 2011. Periodontal ligament and gingival fibroblasts participate in the production of TGF-beta, interleukin (IL)-8 and IL-10. *Braz Oral Res* 25: 157-162.
42. Murphy, K. E., S. W. McCue, and D. L. McElwain. 2012. Clinical strategies for the alleviation of contractures from a predictive mathematical model of dermal repair. *Wound Repair Regen* 20: 194-202.
43. Huang, M., S. Sharma, L. X. Zhu, M. P. Keane, J. Luo, L. Zhang, M. D. Burdick, Y. Q. Lin, M. Dohadwala, B. Gardner, R. K. Batra, R. M. Strieter, and S. M. Dubinett. 2002. IL-7 inhibits fibroblast TGF-beta production and signaling in pulmonary fibrosis. *J Clin Invest* 109: 931-937.
44. Wallace, D. G., and A. Thompson. 1983. Description of collagen fibril formation by a theory of polymer crystallization. *Biopolymers* 22: 1793-1811.
45. Gelman, R. A., B. R. Williams, and K. A. Piez. 1979. Collagen fibril formation. Evidence for a multistep process. *J Biol Chem* 254: 180-186.
46. Hicks, M. R., T. V. Cao, D. H. Campbell, and P. R. Standley. 2012. Mechanical strain applied to human fibroblasts differentially regulates skeletal myoblast differentiation. *J Appl Physiol (1985)* 113: 465-472.
47. Takahashi, M., C. Galligan, L. Tessarollo, and T. Yoshimura. 2009. Monocyte chemoattractant protein-1 (MCP-1), not MCP-3, is the primary chemokine required for monocyte recruitment in mouse peritonitis induced with thioglycollate or zymosan A. *J Immunol* 183: 3463-3471.
48. Smith, M., and G. Bowlin. Feasibility of electrospun polydioxanone monocyte chemotactic protein-1 (MCP-1) hybrid scaffolds as potential cellular homing devices.
49. Gharaee-Kermani, M., E. M. Denholm, and S. H. Phan. 1996. Costimulation of fibroblast collagen and transforming growth factor beta1 gene expression by monocyte chemoattractant protein-1 via specific receptors. *J Biol Chem* 271: 17779-17784.
50. Xiong, M., G. Elson, D. Legarda, and S. J. Leibovich. 1998. Production of vascular endothelial growth factor by murine macrophages: regulation by hypoxia, lactate, and the inducible nitric oxide synthase pathway. *Am J Pathol* 153: 587-598.

51. Lerman, O. Z., R. D. Galiano, M. Armour, J. P. Levine, and G. C. Gurtner. 2003. Cellular dysfunction in the diabetic fibroblast: impairment in migration, vascular endothelial growth factor production, and response to hypoxia. *Am J Pathol* 162: 303-312.
52. Finley, S. D., M. O. Engel-Stefanini, P. I. Imoukhuede, and A. S. Popel. 2011. Pharmacokinetics and pharmacodynamics of VEGF-neutralizing antibodies. *BMC Syst Biol* 5: 193.
53. Schubert, S. Y., A. Benarroch, J. Monter-Solans, and E. R. Edelman. 2011. Primary monocytes regulate endothelial cell survival through secretion of angiopoietin-1 and activation of endothelial Tie2. *Arterioscler Thromb Vasc Biol* 31: 870-875.
54. Blasi, A., C. Martino, L. Balducci, M. Saldarelli, A. Soleti, S. E. Navone, L. Canzi, S. Cristini, G. Invernici, E. A. Parati, and G. Alessandri. 2011. Dermal fibroblasts display similar phenotypic and differentiation capacity to fat-derived mesenchymal stem cells, but differ in anti-inflammatory and angiogenic potential. *Vasc Cell* 3: 5.
55. Jackson, T., and X. Zheng. 2010. A cell-based model of endothelial cell migration, proliferation and maturation during corneal angiogenesis. *Bull Math Biol* 72: 830-868.
56. Jaffe, E. A., J. T. Ruggiero, and D. J. Falcone. 1985. Monocytes and macrophages synthesize and secrete thrombospondin. *Blood* 65: 79-84.
57. Jaffe, E. A., J. T. Ruggiero, L. K. Leung, M. J. Doyle, P. J. McKeown-Longo, and D. F. Mosher. 1983. Cultured human fibroblasts synthesize and secrete thrombospondin and incorporate it into extracellular matrix. *Proc Natl Acad Sci U S A* 80: 998-1002.
58. Raugi, G. J., S. M. Mumby, D. Abbott-Brown, and P. Bornstein. 1982. Thrombospondin: synthesis and secretion by cells in culture. *J Cell Biol* 95: 351-354.
59. Rohrs, J. A., C. D. Sulistio, and S. D. Finley. 2016. Predictive model of thrombospondin-1 and vascular endothelial growth factor in breast tumor tissue. *NPJ Syst Biol Appl* 2.
60. Wu, P., H. Yonekura, H. Li, I. Nozaki, Y. Tomono, I. Naito, Y. Ninomiya, and H. Yamamoto. 2001. Hypoxia down-regulates endostatin production by human microvascular endothelial cells and pericytes. *Biochem Biophys Res Commun* 288: 1149-1154.
61. Billy, F., B. Ribba, O. Saut, H. Morre-Trouilhet, T. Colin, D. Bresch, J. P. Boissel, E. Grenier, and J. P. Flandrois. 2009. A pharmacologically based multiscale mathematical

model of angiogenesis and its use in investigating the efficacy of a new cancer treatment strategy. *J Theor Biol* 260: 545-562.

62. Gaffney, E. A., K. Pugh, P. K. Maini, and F. Arnold. 2002. Investigating a simple model of cutaneous wound healing angiogenesis. *J Math Biol* 45: 337-374.

63. Schugart, R. C., A. Friedman, R. Zhao, and C. K. Sen. 2008. Wound angiogenesis as a function of tissue oxygen tension: a mathematical model. *Proc Natl Acad Sci U S A* 105: 2628-2633.

64. Flegg, J. A., D. L. McElwain, H. M. Byrne, and I. W. Turner. 2009. A three species model to simulate application of Hyperbaric Oxygen Therapy to chronic wounds. *PLOS Comput Biol* 5: e1000451.

65. Abraham, N. G., T. Kushida, J. McClung, M. Weiss, S. Quan, R. Lafaro, Z. Darzynkiewicz, and M. Wolin. 2003. Heme oxygenase-1 attenuates glucose-mediated cell growth arrest and apoptosis in human microvessel endothelial cells. *Circ Res* 93: 507-514.

66. Yuan, P., Y. Bo, G. Ming, F. Wen-Jun, Z. Qin, and H. Bo. 2014. Apoptosis of human umbilical vein endothelial cells (HUVEC) induced by IgA1 isolated from Henoch-Schonlein purpura children. *Asian Pac J Allergy Immunol* 32: 34-38.

67. Hubbard, N. E., D. Lim, M. Mukutmoni, A. Cai, and K. L. Erickson. 2005. Expression and regulation of murine macrophage angiopoietin-2. *Cell Immunol* 234: 102-109.

68. Cheng, S. S., N. W. Lukacs, and S. L. Kunkel. 2002. Eotaxin/CCL11 suppresses IL-8/CXCL8 secretion from human dermal microvascular endothelial cells. *J Immunol* 168: 2887-2894.

69. Bianchi, A., K. J. Painter, and J. A. Sherratt. 2015. A mathematical model for lymphangiogenesis in normal and diabetic wounds. *J Theor Biol* 383: 61-86.

70. Soderquist, B., J. Kallman, H. Holmberg, T. Vikerfors, and E. Kihlstrom. 1998. Secretion of IL-6, IL-8 and G-CSF by human endothelial cells in vitro in response to Staphylococcus aureus and staphylococcal exotoxins. *APMIS* 106: 1157-1164.

71. Famulla, S., D. Lamers, S. Hartwig, W. Passlack, A. Horrighs, A. Cramer, S. Lehr, H. Sell, and J. Eckel. 2011. Pigment epithelium-derived factor (PEDF) is one of the most abundant proteins secreted by human adipocytes and induces insulin resistance and inflammatory signaling in muscle and fat cells. *Int J Obes (Lond)* 35: 762-772.

72. Chen, L., and L. A. DiPietro. 2014. Production and function of pigment epithelium-derived factor in isolated skin keratinocytes. *Exp Dermatol* 23: 436-438.
73. Bai, Y. J., L. Z. Huang, X. L. Xu, W. Du, A. Y. Zhou, W. Z. Yu, and X. X. Li. 2012. Polyethylene glycol-modified pigment epithelial-derived factor: new prospects for treatment of retinal neovascularization. *J Pharmacol Exp Ther* 342: 131-139.
74. Wang, Z., Y. Wang, F. Farhangfar, M. Zimmer, and Y. Zhang. 2012. Enhanced keratinocyte proliferation and migration in co-culture with fibroblasts. *PLOS ONE* 7: e40951.
75. Menon, S. N., J. A. Flegg, S. W. McCue, R. C. Schugart, R. A. Dawson, and D. L. McElwain. 2012. Modelling the interaction of keratinocytes and fibroblasts during normal and abnormal wound healing processes. *Proc Biol Sci* 279: 3329-3338.
76. Gron, B., K. Stoltze, A. Andersson, and E. Dabelsteen. 2002. Oral fibroblasts produce more HGF and KGF than skin fibroblasts in response to co-culture with keratinocytes. *APMIS* 110: 892-898.
77. Rex, J., U. Albrecht, C. Ehlting, M. Thomas, U. M. Zanger, O. Sawodny, D. Haussinger, M. Ederer, R. Feuer, and J. G. Bode. 2016. Model-Based Characterization of Inflammatory Gene Expression Patterns of Activated Macrophages. *PLOS Comput Biol* 12: e1005018.
78. Datta, S., R. Biswas, M. Novotny, P. G. Pavicic, Jr., T. Herjan, P. Mandal, and T. A. Hamilton. 2008. Tristetraprolin regulates CXCL1 (KC) mRNA stability. *J Immunol* 180: 2545-2552.
79. Schiefelbein, D., O. Seitz, I. Goren, J. P. Dissmann, H. Schmidt, M. Bachmann, R. Sader, G. Geisslinger, J. Pfeilschifter, and S. Frank. 2008. Keratinocyte-derived vascular endothelial growth factor biosynthesis represents a pleiotropic side effect of peroxisome proliferator-activated receptor-gamma agonist troglitazone but not rosiglitazone and involves activation of p38 mitogen-activated protein kinase: implications for diabetes-impaired skin repair. *Mol Pharmacol* 74: 952-963.
80. Fang, W. B., B. Mafuvadze, M. Yao, A. Zou, M. Portsche, and N. Cheng. 2015. TGF-beta Negatively Regulates CXCL1 Chemokine Expression in Mammary Fibroblasts through Enhancement of Smad2/3 and Suppression of HGF/c-Met Signaling Mechanisms. *PLOS ONE* 10: e0135063.

81. De Crescenzo, G., P. L. Pham, Y. Durocher, and M. D. O'Connor-McCourt. 2003. Transforming growth factor-beta (TGF-beta) binding to the extracellular domain of the type II TGF-beta receptor: receptor capture on a biosensor surface using a new coiled-coil capture system demonstrates that avidity contributes significantly to high affinity binding. *J Mol Biol* 328: 1173-1183.

82. Brandes, M. E., U. E. Mai, K. Ohura, and S. M. Wahl. 1991. Type I transforming growth factor-beta receptors on neutrophils mediate chemotaxis to transforming growth factor-beta. *J Immunol* 147: 1600-1606.

83. Wahl, S. M., D. A. Hunt, L. M. Wakefield, N. McCartney-Francis, L. M. Wahl, A. B. Roberts, and M. B. Sporn. 1987. Transforming growth factor type β induces monocyte chemotaxis and growth factor production. *Proc Natl Acad Sci U S A* 84: 5788-5792.

84. Postlethwaite, A. E., J. Keski-Oja, H. L. Moses, and A. H. Kang. 1987. Stimulation of the chemotactic migration of human fibroblasts by transforming growth factor beta. *J Exp Med* 165: 251-256.

85. Metzger, W., N. Grenner, S. E. Motsch, R. Strehlow, T. Pohlemann, and M. Oberringer. 2007. Induction of myofibroblastic differentiation in vitro by covalently immobilized transforming growth factor-beta(1). *Tissue Eng* 13: 2751-2760.

86. Deuel, T. F., R. M. Senior, J. S. Huang, and G. L. Griffin. 1982. Chemotaxis of monocytes and neutrophils to platelet-derived growth factor. *J Clin Invest* 69: 1046-1049.

87. Senior, R. M., G. L. Griffin, J. S. Huang, D. A. Walz, and T. F. Deuel. 1983. Chemotactic activity of platelet alpha granule proteins for fibroblasts. *J Cell Biol* 96: 382-385.

88. Pai, R., H. Ha, M. A. Kirschenbaum, and V. S. Kamanna. 1996. Role of tumor necrosis factor-alpha on mesangial cell MCP-1 expression and monocyte migration: mechanisms mediated by signal transduction. *J Am Soc Nephrol* 7: 914-923.

89. Postlethwaite, A. E., and J. M. Seyer. 1990. Stimulation of fibroblast chemotaxis by human recombinant tumor necrosis factor alpha (TNF-alpha) and a synthetic TNF-alpha 31-68 peptide. *J Exp Med* 172: 1749-1756.

90. Orfanos, S. E., A. Kotanidou, C. Glynos, C. Athanasiou, S. Tsigkos, I. Dimopoulou, C. Sotiropoulou, S. Zakynthinos, A. Armaganidis, A. Papapetropoulos, and C. Roussos. 2007. Angiopoietin-2 is increased in severe sepsis: correlation with inflammatory mediators. *Crit Care Med* 35: 199-206.

91. Yao, P., A. A. Potdar, P. S. Ray, S. M. Eswarappa, A. C. Flagg, B. Willard, and P. L. Fox. 2013. The HILDA complex coordinates a conditional switch in the 3'-untranslated region of the VEGFA mRNA. *PLOS Biol* 11: e1001635.
92. Wang, J. M., B. Sherry, M. J. Fivash, D. J. Kelvin, and J. J. Oppenheim. 1993. Human recombinant macrophage inflammatory protein-1 alpha and -beta and monocyte chemotactic and activating factor utilize common and unique receptors on human monocytes. *J Immunol* 150: 3022-3029.
93. Grant, M. B., P. T. Khaw, G. S. Schultz, J. L. Adams, and R. W. Shimizu. 1992. Effects of epidermal growth factor, fibroblast growth factor, and transforming growth factor-beta on corneal cell chemotaxis. *Invest Ophthalmol Vis Sci* 33: 3292-3301.
94. Tsukamoto, Y., W. E. Helsel, and S. M. Wahl. 1981. Macrophage production of fibronectin, a chemoattractant for fibroblasts. *J Immunol* 127: 673-678.
95. Uguccioni, M., M. D'Apuzzo, M. Loetscher, B. Dewald, and M. Baggiolini. 1995. Actions of the chemotactic cytokines MCP-1, MCP-2, MCP-3, RANTES, MIP-1 alpha and MIP-1 beta on human monocytes. *Eur J Immunol* 25: 64-68.
96. Bernatchez, P. N., S. Soker, and M. G. Sirois. 1999. Vascular endothelial growth factor effect on endothelial cell proliferation, migration, and platelet-activating factor synthesis is Flk-1-dependent. *J Biol Chem* 274: 31047-31054.
97. Yoo, M. H., H. J. Hyun, J. Y. Koh, and Y. H. Yoon. 2005. Riluzole inhibits VEGF-induced endothelial cell proliferation in vitro and hyperoxia-induced abnormal vessel formation in vivo. *Invest Ophthalmol Vis Sci* 46: 4780-4787.
98. Gerber, H. P., A. McMurtrey, J. Kowalski, M. Yan, B. A. Keyt, V. Dixit, and N. Ferrara. 1998. Vascular endothelial growth factor regulates endothelial cell survival through the phosphatidylinositol 3'-kinase/Akt signal transduction pathway. Requirement for Flk-1/KDR activation. *J Biol Chem* 273: 30336-30343.
99. Man, X. Y., X. H. Yang, S. Q. Cai, Y. G. Yao, and M. Zheng. 2006. Immunolocalization and expression of vascular endothelial growth factor receptors (VEGFRs) and neuropilins (NRPs) on keratinocytes in human epidermis. *Mol Med* 12: 127-136.
100. Abdel-Malak, N. A., C. B. Srikant, A. S. Kristof, S. A. Magder, J. A. Di Battista, and S. N. Hussain. 2008. Angiopoietin-1 promotes endothelial cell proliferation and migration through AP-1-dependent autocrine production of interleukin-8. *Blood* 111: 4145-4154.

101. Murdoch, C., S. Tazzyman, S. Webster, and C. E. Lewis. 2007. Expression of Tie-2 by human monocytes and their responses to angiopoietin-2. *J Immunol* 178: 7405-7411.

102. Longeras, R., K. Farjo, M. Ihnat, and J. X. Ma. 2012. A PEDF-derived peptide inhibits retinal neovascularization and blocks mobilization of bone marrow-derived endothelial progenitor cells. *Exp Diabetes Res* 2012: 518426.

103. Zhang, S. X., J. J. Wang, G. Gao, K. Parke, and J. X. Ma. 2006. Pigment epithelium-derived factor downregulates vascular endothelial growth factor (VEGF) expression and inhibits VEGF-VEGF receptor 2 binding in diabetic retinopathy. *J Mol Endocrinol* 37: 1-12.

104. Siddiqui, A., R. D. Galiano, D. Connors, E. Gruskin, L. Wu, and T. A. Mustoe. 1996. Differential effects of oxygen on human dermal fibroblasts: acute versus chronic hypoxia. *Wound Repair Regen* 4: 211-218.

105. Utting, J. C., S. P. Robins, A. Brandao-Burch, I. R. Orriss, J. Behar, and T. R. Arnett. 2006. Hypoxia inhibits the growth, differentiation and bone-forming capacity of rat osteoblasts. *Exp Cell Res* 312: 1693-1702.

106. van Vlimmeren, M. A., A. Driessen-Mol, M. van den Broek, C. V. Bouten, and F. P. Baaijens. 2010. Controlling matrix formation and cross-linking by hypoxia in cardiovascular tissue engineering. *J Appl Physiol (1985)* 109: 1483-1491.

107. Notari, L., A. Miller, A. Martinez, J. Amaral, M. Ju, G. Robinson, L. E. Smith, and S. P. Becerra. 2005. Pigment epithelium-derived factor is a substrate for matrix metalloproteinase type 2 and type 9: implications for downregulation in hypoxia. *Invest Ophthalmol Vis Sci* 46: 2736-2747.

108. O'Reilly, M. S., T. Boehm, Y. Shing, N. Fukai, G. Vasios, W. S. Lane, E. Flynn, J. R. Birkhead, B. R. Olsen, and J. Folkman. 1997. Endostatin: an endogenous inhibitor of angiogenesis and tumor growth. *Cell* 88: 277-285.

109. Short, S. M., A. Derrien, R. P. Narsimhan, J. Lawler, D. E. Ingber, and B. R. Zetter. 2005. Inhibition of endothelial cell migration by thrombospondin-1 type-1 repeats is mediated by beta1 integrins. *J Cell Biol* 168: 643-653.

110. Le Bellego, F., H. Perera, S. Plante, J. Chakir, Q. Hamid, and M. S. Ludwig. 2009. Mechanical strain increases cytokine and chemokine production in bronchial fibroblasts from asthmatic patients. *Allergy* 64: 32-39.

111. Yang, G., R. C. Crawford, and J. H. Wang. 2004. Proliferation and collagen production of human patellar tendon fibroblasts in response to cyclic uniaxial stretching in serum-free conditions. *J Biomech* 37: 1543-1550.

112. Okizaki, S., Y. Ito, K. Hosono, K. Oba, H. Ohkubo, K. Kojo, N. Nishizawa, M. Shibuya, M. Shichiri, and M. Majima. 2016. Vascular Endothelial Growth Factor Receptor Type 1 Signaling Prevents Delayed Wound Healing in Diabetes by Attenuating the Production of IL-1beta by Recruited Macrophages. *Am J Pathol* 186: 1481-1498.

113. Mirza, R., L. A. DiPietro, and T. J. Koh. 2009. Selective and specific macrophage ablation is detrimental to wound healing in mice. *Am J Pathol* 175: 2454-2462.

114. Altavilla, D., A. Saitta, D. Cucinotta, M. Galeano, B. Deodato, M. Colonna, V. Torre, G. Russo, A. Sardella, G. Urna, G. M. Campo, V. Cavallari, G. Squadrito, and F. Squadrito. 2001. Inhibition of lipid peroxidation restores impaired vascular endothelial growth factor expression and stimulates wound healing and angiogenesis in the genetically diabetic mouse. *Diabetes* 50: 667-674.

115. Kampfer, H., J. Pfeilschifter, and S. Frank. 2001. Expressional regulation of angiopoietin-1 and -2 and the tie-1 and -2 receptor tyrosine kinases during cutaneous wound healing: a comparative study of normal and impaired repair. *Lab Invest* 81: 361-373.
