# Supplementary material for: Predictive Approach Identifies Molecular Targets and Interventions to Restore Angiogenesis in Wounds With Delayed Healing
Source: Front Physiol. 2019 May 28;10:636. doi: 10.3389/fphys.2019.00636 (PMC6547939; doi:10.3389/fphys.2019.00636)
Supplement: Supplementary file 2 [file Data_Sheet_2.zip › AngiogenesisMATLAB code/MATLAB code manual.pdf]

MATLAB Code Description

16 May 2019

Predictive approach identifies molecular targets and interventions to restore angiogenesis in wounds with delayed healing

by Sridevi Nagaraja, Lin Chen, Luisa A. DiPietro, Jaques Reifman, and Alexander Y. Mitrophanov

Correspondence about modeling- and software-related technical questions to Sridevi Nagaraja: snagaraja@bhsai.org

CONTENTS

Overview..3
System Requirements 3
MATLAB Code Description 4
Normal and impaired angiogenesis simulations...5
Extended Sensitivity Analysis (randomized parameter sets).............................6
Correlation Analysis (randomized parameter sets)..7
Protein-knockout analysis...8
Intervention strategy simulations...8

Overview

The MATLAB code for the angiogenesis model was developed at the Biotechnology High Performance Computing Software Applications Institute (BHSAI), Ft. Detrick, Maryland, to identify influential molecular and cellular processes, as well as protein targets, whose modulation may stimulate angiogenesis in wounds. The model is further used to investigate intervention strategies involving the modulation of the model-identified molecular targets to restore angiogenesis in delayed-healing wounds. The software currently implements a set of 39 ordinary differential equations (ODEs) and a delay differential equation (DDE) to simulate normal and impaired angiogenesis. Sensitivity and correlation analyses were implemented to identify the key processes that were influential for wound angiogenesis. A protein-knockout analysis was used to identify influential molecular targets. The inputs for the angiogenesis model as well as the sensitivity analyses are embedded in the code. The user can directly change parameter values inside some of the provided MATLAB files. The System Requirements Section contains the details about the computer system that we used to develop and run the code. The rest of this document provides information about using the code for the three different types of analysis described in the paper (modeling normal and impaired angiogenesis scenarios, as well as sensitivity, correlation, and protein-knockout analyses, and simulating intervention strategies).

System Requirements

We used the following software and hardware components:

Software

- Operating System: Windows 7 Enterprise (64-bit operating system)
- MATLAB version 9.3.0.713579 (R2017b) (64-bit operating system)
- MATLAB Statistics Toolbox, Version 8.0 (R2017b)
- Microsoft Excel 2016 for plotting the figures in the paper

Hardware

- Intel® Core(TM) 2Duo CPU E8400 @ 3.00 GHz and 4.00 GB RAM
- Disk space: 3–4 GB is recommended for a typical installation

For extended sensitivity analysis, we used two server computers with the following specifications:

- CPU: 2x Intel Xeon X5650 (6 cores @ 2.66 GHz)
- RAM: 24 GB @ 1333 MHz
- Disk space: 4x 300 GB @ 10,000 RPM

MATLAB Code Description

The code was developed in MATLAB 2017b. It includes the following files:

**Main.m**	–	simulation routine that runs the default model plots the model outputs time courses during normal angiogenesis
**Main_timeinhibition.m**	–	simulation routine that runs the TGF-β inhibition routine with inhibitor introduction at different user specified time points
**woundhealingequations2.m**	–	function comprising model equations, as well as chemotaxis and cytokine feedback functions
**woundhealingequations2_inhibition.m**	–	function comprising the additional equations to represent TGF-β inhibitor
**Parameters.m**	–	script containing parameter values and initial conditions (also contains the program's main INPUT)
**Parameters_inhibition.m**	–	script containing parameter values and initial conditions including activation and deactivation rate constants for the TGF-β inhibitor
**Graphs_main.m**	–	script that performs basic plotting of all model variables
**Global_local.m**	–	simulation routine for extended sensitivity analysis (in the vicinities of 60,000 random parameter sets)
**Latinhypercube.m**	–	function that generates a user-defined number of random parameter sets (60,000 in this study)
**Curvecharacteristic.m**	–	function for calculating the logarithmic local sensitivity values for each model output variable for a given parameter set
**Main_analysis.m**	–	simulation routine for performing the extended sensitivity analysis and the PRCC analysis
**errorremovedmatrix.m**	–	function to remove erroneous simulations that failed to converge during the global sensitivity analysis
**top5ranks**	–	function to extract the 5 highest sensitivity values for the outputs of interest (EC and VEGF in this study)

ranksorting.m – function to sort and count the top 5 sensitivity indices for EC and VEGF model outputs across 60,000 simulations

PRCC2.m – function for calculating the Spearman's partial correlation coefficient and their respective p-values between the 60,000 parameter values and 60,000 values of each model output variable at 40 different time points

analysis_SA.m – script for separating the 60,000 simulations into normal and impaired cases and performing the protein-knockout analysis

timescalculation.m – function for calculating the four inflammatory indices for all model variables

knockoutcalculation.m – function for calculating the four indices and time courses of the model output variables after the knockout of a specific protein

Instructions for downloading and saving the MATLAB files

The files are currently available in a zip folder that can be downloaded from the Frontiers in Physiology website. In order to run them in MATLAB, please follow the instructions given below:
1. Download the zipped folder Angiogenesis MATLAB Code onto your system.
2. Unzip the folder and save all the files into a new folder.
3. Open folder created in step 2 in MATLAB as your current working folder.

Normal and impaired angiogenesis simulations

1. The **INPUT** to the model is the **initial concentration of platelets** which reflects the severity of an injury. The default value of this parameter is **2×10^8 platelets/mL**. This value is defined in the **"Parameters.m"** file under **"Initial conditions"**. To increase or decrease the severity of an injury, increase or decrease the value of the parameter **"P_init"** in this file.

Note: Any additional simulation of interest, e.g., impaired angiogenesis induced by a 1.2 fold higher endothelial cell apoptosis rate, will need to be initiated by changing the respective parameter in the **"Parameters.m"** file.

2. To run the model, open "**Main.m"** and click the **"Run"** icon 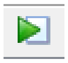 or type **"Main"** in the MATLAB command window.

3. After the routine is executed, to display all model variables run **“Graphs_main.m”**.

4. These output figures show the raw output values calculated by the model. The raw values of all output variables, as well as simulation time points, are stored in the output variable **“g”** in the MATLAB workspace.

Note: The raw values of all output variables of the model were imported into Microsoft (MS) EXCEL, normalized, before being used in the figures in the manuscript.

Note: Impaired angiogenesis simulations shown in Figs. 3, 6, and 7 of the paper were performed by executing the file **“Main.m”** after increasing the endothelial cell apoptosis rate parameter **“kapop_EC”** by 1.2 fold and decreasing the VEGF production rate by anti-inflammatory macrophages parameter **“kVEGF_anti”** by 3 fold of their default values in the **“Parameters.m”** file.

Extended Sensitivity Analysis (randomized parameter sets)

Simulation: To calculate sensitivity values for a number of random parameter sets, follow the instructions given below:

1. Open **“Global_local.m”**.

2. Change the value of the parameter **“iter”** to choose the number of randomly generated parameter sets (default value used in the paper: 60,000).

3. Change the value of the parameter **“rangefactor”** to increase or decrease the uniform distribution sampling range for the extended sensitivity analysis [default value used in the paper: 2.5, i.e., the parameter values in the generated sets are chosen randomly from a 5-fold range (2.5-fold higher and 2.5-fold lower than default value)].

Note: To perform the sensitivity analysis on one parameter set using one node takes approximately 5 minutes. Therefore, to compute the sensitivity values for 60,000 parameter sets, we used 12 parallel nodes, and the simulation took approximately 16 hours to complete. The user is recommended to start with smaller values of **“iter”** (e.g., 50 or 100). User can specify the number of nodes that will be used in **“parpool”** command right before using **“parfor”.** If parallel processing is not being used, replace the **“parfor”** command in **“Global_local.m”**, line 47, by **“for”** and comment lines 43-44, lines 60, 69, and 72-73.

4. Click on the **“Run”** icon or type **“Global_local”** in the MATLAB command window.

Output: The raw sensitivities for each of the 40 model variables to each of the 159 model parameters at 40 time points of the simulation. The size of each of these matrices is 40 x 60,000. These sensitivity values are stored in the output variable **"G_global"**. The values of the model parameters in the 60,000 sets are stored in the output variable **"Param1"**. This routine also calculates the four inflammatory indices (activation time, peak height, resolution interval, and resolution plateau) from the time courses of each model output variables in the 60,000 simulations. The values of these indices are stored in **"ATf"**, **"PHf"**, **"DTf"**, and **"RPf"**, respectively. These five variables mentioned immediately above are saved together in one output variable called **"outputvars.mat".** The individual time courses of each model output variable in the 60,000 simulations are stored in the output variable **"Y_main_global"** and are further used (along with the parameter values stored in **"Param1"**) for the calculation of the Spearman's partial rank correlation coefficient between the model parameters and the model output variables. The user may individually save the output variables once the run is complete.

Note: In this routine, the simulations run using some of the 60,000 parameter sets failed to converge. When time happened, a default value was saved for the output variables and sensitivities for that particular run and the specific number of that parameter set (from between 1 to 60,000) was stored in the output variable **"E"**. These parameter sets and the corresponding simulations in **"E"** were not used for any other analyses.

5. To perform the extended local sensitivity analysis, open **"Main_analysis.m"**.

6. Run the section of the code for extended sensitivity analysis

Output: The output provides the number of the model parameter (each parameter is assigned a number, see Supplemental Table S2) that the model outputs EC and VEGF were most sensitive to in the majority of the 60,000 simulations in **"H1.mat"**. The exact number of those simulations (out of 60,000) is saved in **"H2.mat".**

Note: For a complete list of the parameter identifying numbers, check the file **"Parameters.m"**. Each parameter is assigned an identifying number shown in the comment next to its initialization and in Supplemental Table S2 of the paper.

Correlation Analysis (randomized parameter sets)

Simulation: To calculate the Spearman's rank correlation coefficient values between each model parameter and each model output variable, follow the instructions given below:

1. Load the saved output variable **"outputvars.mat"** and **"Y_main_global.mat"**, or individually load the saved time courses of all the model output variables calculated during the extended sensitivity analysis into the MATLAB workspace.

2. To perform the correlation analysis, open "Main_analysis.m".

3. Run the section of the code for correlation analysis.

Output: The correlation coefficient values between a given model outputs variable and a model parameter at the specified time points (**"M.prcc.mat"**) along with the sign (positive or negative) of the correlation coefficient (**"M.sign.mat"**). From the loaded variable in the MATLAB space, separate out the values of the model output variables of interest. The model output variable numbers are provided in the **"woundhealingequations2.m"** file.

Note: The raw values of all the correlation coefficients were imported into Microsoft (MS) EXCEL, before being used in the figures in the manuscript (Fig. 4 and Fig S1).

Protein-knockout analysis

Simulation: To calculate the effect of knocking out individual proteins on the peak values of EC and VEGF time courses, follow the instructions given below:

1. Load the saved output variable **"outputvars.mat"** and **"Y_main_global.mat"** calculated and saved after running the file **"Global_local.m"**.

2. Open **"analysis_SA.m"** and click on the **"Run"** icon or type **"analysis_SA"** in the MATLAB command window.

Note: In our simulation, we only check the effect of protein knockout on two model output variables representing the EC and VEGF. To check the effect of protein knockouts on other outputs, please add or remove the values in the variable **"P"** and **"Pname"** in **"analysis_SA.m"**. The number of each model output variable can be found in **"woundhealingequations2.m"**.

Intervention strategy simulations

Simulation: Two types of simulations were performed with respect to molecular mediator manipulation to restore angiogenesis.

a) Varying the concentration of the TGF-β inhibitor introduced in the system at time (1h or 24h) at a concentration of 20nM, 50 nM, or 100 nM

b) Adding the molecular mediators FGF-2 (at concentrations 0.1 and 0.05 ng/mL) and ANG-2 (0.05 and 0.015 ng/mL) in the system at time 1h or 24h

In the paper, all the simulations with addition of TGF-β inhibitor, FGF-2, and ANG-2 are simulated along with the parameter changes that simulate impaired angiogenesis in the model.

<u>a) Instructions for running the code for varying the molecular mediator inhibitor concentration</u>

1. Open the **“Parameters_inhibition.m”** file and introduce the following changes.

2. To introduce the individual/combined modulation of a particular molecular mediator in the model, change its/their initial value(s) in the **“Parameters_inhibition.m”** (Itgf_init, kadd_FGF, or kadd_ANG-2).

3. Save the file with the changed model parameter values in steps 1 and 2.

4. Open the file **“Main_inhibition.m”**. In order to introduce the TGF-β inhibitor at a later time point during the simulation, change the value of the parameter **“dosetime”** in the **“Main_inhibiton.m”** file.

5. Click on the **“Run”** icon or type **“Main_inhibition”** in the MATLAB command window.

Output: The time courses of all model variables are stored in the output variable **“Y”** in the MATLAB workspace.

Note: The raw values of all output variables of the model were imported into Microsoft (MS) EXCEL, normalized, before being used in the figures and tables in the manuscript.
